# Consequences of changing Canadian activity patterns since the COVID-19 pandemic include increased residential radon gas exposure for younger people

**DOI:** 10.1038/s41598-023-32416-8

**Published:** 2023-04-07

**Authors:** Natasha L. Cholowsky, Myra J. Chen, Ghozllane Selouani, Sophie C. Pett, Dustin D. Pearson, John M. Danforth, Shelby Fenton, Ela Rydz, Matthew J. Diteljan, Cheryl E. Peters, Aaron A. Goodarzi

**Affiliations:** 1 Robson DNA Science Centre, Department of Biochemistry and Molecular Biology, Charbonneau Cancer Institute, Cumming School of Medicine, University of Calgary, Calgary, AB Canada; 2 Department of Oncology, Charbonneau Cancer Institute, Cumming School of Medicine, University of Calgary, Calgary, AB Canada; 3 Glacier Communications, Inc., Calgary, AB Canada; 4 British Columbia Centre for Disease Control, British Columbia Cancer, School of Population and Public Health, University of British Columbia, Vancouver, BC Canada

**Keywords:** Environmental impact, Psychology and behaviour, Cancer prevention

## Abstract

The COVID-19 pandemic has produced widespread behaviour changes that shifted how people split their time between different environments, altering health risks. Here, we report an update of North American activity patterns before and after pandemic onset, and implications to radioactive radon gas exposure, a leading cause of lung cancer. We surveyed 4009 Canadian households home to people of varied age, gender, employment, community, and income. Whilst overall time spent indoors remained unchanged, time in primary residence increased from 66.4 to 77% of life (+ 1062 h/y) after pandemic onset, increasing annual radiation doses from residential radon by 19.2% (0.97 mSv/y). Disproportionately greater changes were experienced by younger people in newer urban or suburban properties with more occupants, and/or those employed in managerial, administrative, or professional roles excluding medicine. Microinfluencer-based public health messaging stimulated health-seeking behaviour amongst highly impacted, younger groups by > 50%. This work supports re-evaluating environmental health risks modified by still-changing activity patterns.

The 2001 *National Human Activity Pattern Study* demonstrated that the typical, late twentieth century North American adult spent ~ 69% of life (6018 h/y) inside a residential building, and ~ 87% of life indoors generally^1^. The percentage of life split between a primary residence (‘at home’), different residential properties, non-residential buildings (e.g., offices, schools, hotels, factories, shopping centres, etc.), vehicles (cars, buses, trains, etc.), or outside is largely a function of behaviours that differ by age, health status, job type, and other personal and lifestyle variables, and can change seasonally or over a lifetime. Behaviourally driven activity patterns are important determinants of health, since the amount of time that people spend within different buildings determines the duration of exposure to numerous indoor- and outdoor-environmental toxicants that modify disease risk. This is very important in the case of indoor air quality, which can vary substantially between buildings but is generally an unavoidable exposure of occupying (i.e., breathing in) an environment. One of the most prevalent and lethal human diseases driven by exposure to indoor air toxicants is lung cancer, which currently underlies 1 in 4 cancer-related deaths in North America and Europe. In addition to tobacco smoke, lung cancer risk is a function of exposure to carcinogenic triggers such as radioactive radon gas, particulate matter air pollution, arsenic, and asbestos, as well as modifiers such as history of inflammatory lung disease (including pneumonia, tuberculosis, emphysema), diet, fitness, and/or genetics^2–4^.

Radioactive radon (^222^Rn) exposure is an anthropogenic indoor air health problem driven by building practices that have increasingly captured, contained, and concentrated radon to unnaturally high and unsafe levels^5–8^. In North America, residential radon levels have increased over time, with new properties typically being constructed with 72% greater radon levels compared to early to mid twentieth century equivalents^5–8^. Repeated, long term radon inhalation is a primary cause of lung cancer amongst people who have never smoked tobacco^2,3,5,6,9–15^, a disease that is now the 7^th^ leading cause of cancer-linked death globally^9,16–19^. The inhalation of radon and its progeny can increase lung cancer risk as they emit alpha particle radiation within the lungs, which damages lung epithelial cell DNA and thereby increases the risk of cancer-causing mutations^2,20–23^. Radioactivity levels from radon in air are measured in Becquerels (Bq) per cubic meter (m^3^), equal to one alpha particle (radioactive) emission per second per cubic metre of air^2^. Calculating the absorbed radiation doses from radon for an individual involves combining a quantitative measure of Bq/m^3^ radon levels with so-called ‘activity pattern’ data, meaning the amount of time per year a person spends within in a given environment, then deriving Sievert (Sv) doses of absorbed energy per mass. With some exceptions (e.g., twentieth century uranium miners), the majority of lifetime radon exposure occurs within the residential built environment, with the global pre-pandemic average radiation dose rate from residential radon being 1.2 mSv per year (mSv/y), varying by region and demographics^7,13^. For large populations, there is a 16% increase in relative lifetime risk of lung cancer per 100 Bq/m^3^ of radon exposure, equating to 4 mSv/y of absorbed radiation^24,25^.

In addition to the built environment, behaviour and socioeconomics also influence radon exposure^9,26–30^. For example, excess annual radiation doses (from radon) change substantially as a function of how fast individuals gain radon awareness and respond to personal exposure knowledge^26^. Further, as older, more centrally located (relative to an urban core) North American properties have become more expensive within many real estate markets, they have also become less accessible to younger people who are instead more likely to live in newer, relatively more affordable properties with demonstrably higher average radon levels^6–8^. This socioeconomic effect has also biased higher radon exposure towards people with the greatest number of young children living at home^7^, which is particularly concerning as still-growing and replicating human tissue is especially vulnerable to the negative effects of ionizing radiation exposure, including alpha particles from radon^21,31–36^. Historically, this age-related skew has been somewhat offset by the fact that the behaviour of younger adults mean that they are also more likely to be in work, in school and/or more active outside, thus spending less overall time at home compared to older people^26^. We speculate, however, that this paradigm shifted as responses to the COVID-19 pandemic had collateral consequences on behaviour, including altered activity patterns that are unlikely to return to the pre-pandemic status quo. For example, widespread telecommuting has been normalized across many regions, with heightened demand for permanent full or part time work-from-home arrangements now prevalent across many sectors^37,38^. The scale and impact of such changes on the effects of environmental determinants of health, or how to respond to this, are not clear.

The balance in time spent between residential and non-residential environments matters as many North American occupational environments carry lower radon exposure risks due to safety programs in operation for many years, some commencing as early as the 1970s in the mining sector^21,39–41^. Such programs have successfully lowered radon levels within many public and private workplace building portfolios, with notable examples in Canada being all federal and many provincial and municipal government-operated facilities (e.g., administration, national parks, border control, military, national police, prisons, and education)^42–45^. Further to this, ventilation controls in buildings such as office towers, hospitals, retail malls, hotels, factories, etc. typically operate with greater air exchanges and/or positive pressure versus a typical residential property, and so are more likely to produce indoor air with lower overall radon risk^42,46^. Measuring shifts in the relative amount of time per year between environments with differing environmental carcinogen risks is critical to understand future cancer burdens. To help address this, we assessed the magnitude of human activity pattern shifts since the onset of the COVID-19 pandemic in Canada, how these changes differed across diverse groups of people, to what extent changes altered residential radon exposure, and also evaluated the efficacy of a communication strategy aimed at enhancing health-seeking behaviour in those most impacted.

## Results

### Demographic characteristics of the human activity pattern cohort

**Figure 1:**
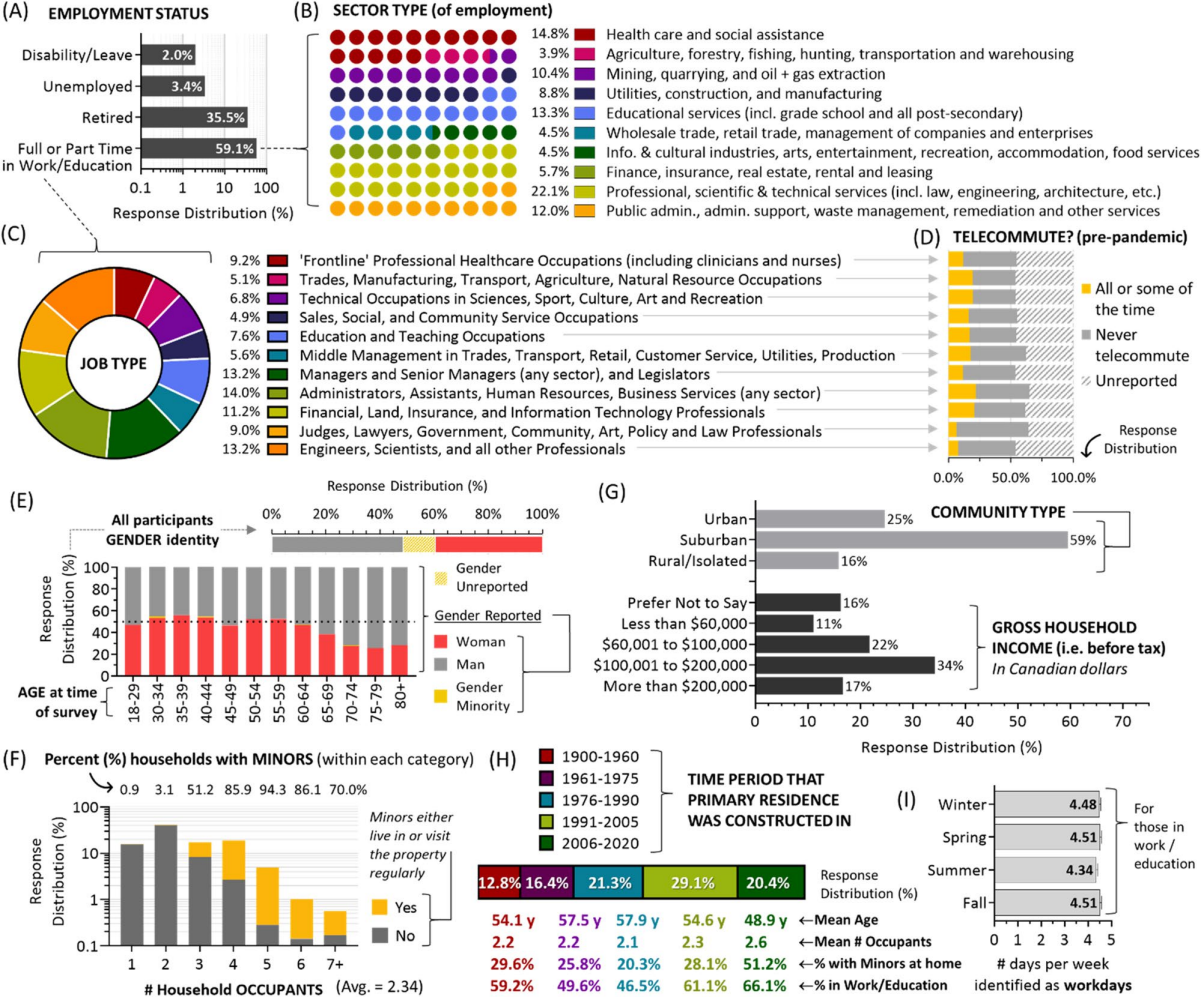
Demographic characteristics of the human activity pattern study cohort. Panel **A**. Study cohort by employment status. Panel **B**. Sector of employment for those in full or part time work, where each colour corresponds to the indicated sector, and each circle represents 1% of the total cohort. Panel **C**: A pie chart showing the distribution of the indicated job types for those in full or part time work. Panel **D**. Reported telecommuting during the pre-pandemic period for those in work, as a function of job type. Panel **E**. Top graph, the distribution of reported and unreported gender identities. Bottom graph, for those of known gender, distribution by age bracket. Panel **F**. Distribution by number of household occupants, with yellow bars indicating percentage where minors either live full time or visit regularly. Panel **G**. Breakdown by community identity and gross household income bracket. Panel **H**. Distribution of primary residences by year of construction bracket, coupled with geometric mean age, occupant number, % minors at home and % in work or education by grouping. Panel **I**. Number of days identified by those in full or part time employment (or education) as workdays, by season. Figures were prepared using Excel and GraphPad Prism 9.1.1 (225) (www.graphpad.com).

The total study population included all adults enrolled in the Canadian ‘Evict Radon National Study’, a publicly funded radon testing, human behaviour, and knowledge translation project led by investigators at multiple universities. Of these, 19,441 households active within the study in 2021 were invited to provide detailed human activity patterns and demographic information, with all adult occupiers of any residential building type being equally eligible for study inclusion, irrespective of whether they owned or rented the property. Informed consent and online survey responses from primary respondents in 4009 households (with 9785 occupants) were obtained, in English or French, with survey questions described in Supplemental Information, Section I. Overall, 59.1% of primary respondents were in full or part time work or education, 35.5% were retired, 3.4% were unemployed, and 2% were on short term (e.g., parental) or long term (e.g., disability) leave (Fig. 1A). Those in full or part time work were employed across diverse sectors (Fig. 1B) and in multiple job types (Fig. 1C) spanning the entirety of the North American sector and job code index (see methods). Depending on job type, between 6.4 to 21.5% of those in full or part time work reported telecommuting at least some or all of the time prior to the onset of the COVID-19 pandemic (Fig. 1D). Overall, 12% declined to report their gender, 48% identified as cisgender men, 40% as cisgender women, and 0.1% as a gender minority (Fig. 1E). For those of known gender identity, the cohort was balanced between men and women aged 18–64, and somewhat over-represented by men amongst those aged 65 +. The geometric mean age was 53.9 y, which was comparable to the adult population for this region at the time of surveying (50.3 y)^47^. The average number of household occupants was 2.34 (regional average = 2.47)^47^, with 51.2–94.3% of households with 3 + occupants reporting that minors (ages 0–17) lived in or visited the property regularly (Fig. 1F). A majority (59%) of households identified their community as suburban, with 25% being urban, and 16% being rural or in an isolated area **(**Fig. 1G). Gross (i.e., before tax) household income was reported by 84% of participants and reflected regional averages (CAD$118,000)^47^, with some skew towards middle income earners (Fig. 1G). Primary residences were constructed from 1900 to 2020, with newer properties containing comparatively younger people, more household occupants, more minors, and people more likely to be in work or education (Fig. 1H).

### Human activity patterns before and after the onset of the COVID-19 pandemic

**Figure 2:**
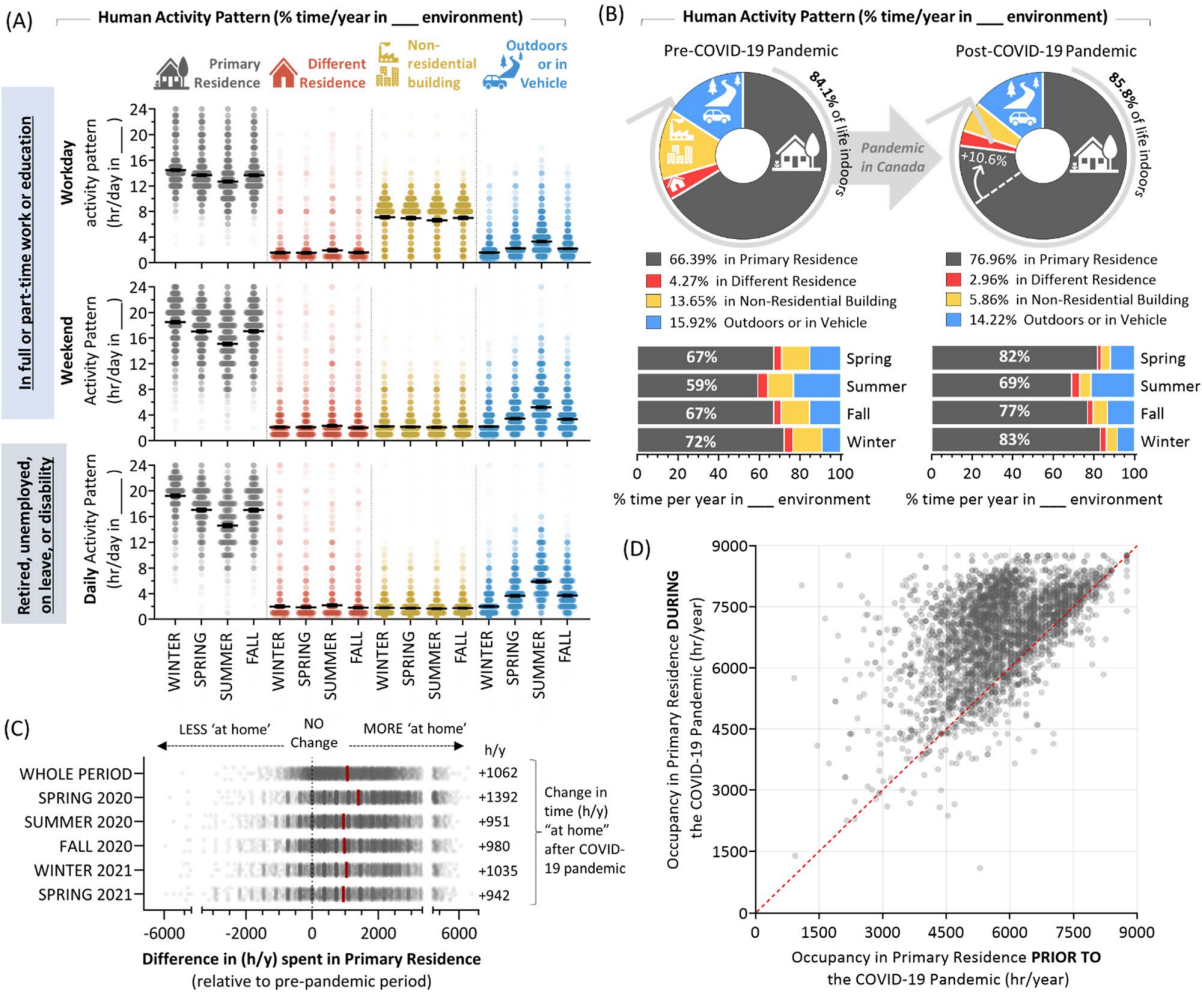
Canadian activity patterns before and after the onset of the COVID-19 pandemic. Panel **A**. For the pre-pandemic period, the number of hours per day spent across different environments including the primary residence (grey), a different residential building (red), any non-residential building type (yellow), or in a vehicle or outside (blue), as a function of seasons. Bars represent arithmetic means. For those in work or education, workdays were distinguished from weekends (including holidays). Panel **B**. Using the data in (**A**), the percent of time per year in each environment for the entire year (pie chart), or by season (bar charts) for the pre-COVID-19 pandemic period (a typical year prior to March 2020), and post-COVID-19 pandemic periods. Panel **C**. The absolute difference in hours spent in the primary residence relative to the pre-pandemic period. Red bars represent arithmetic mean h/y differences. Panel **D**. Scatter plot showing h/y that individuals spent in their primary residence prior to (x-axis) or during (y-axis) the COVID-19 pandemic. Figures were prepared using Excel and GraphPad Prism 9.1.1 (225) (www.graphpad.com).

**Figure 3:**
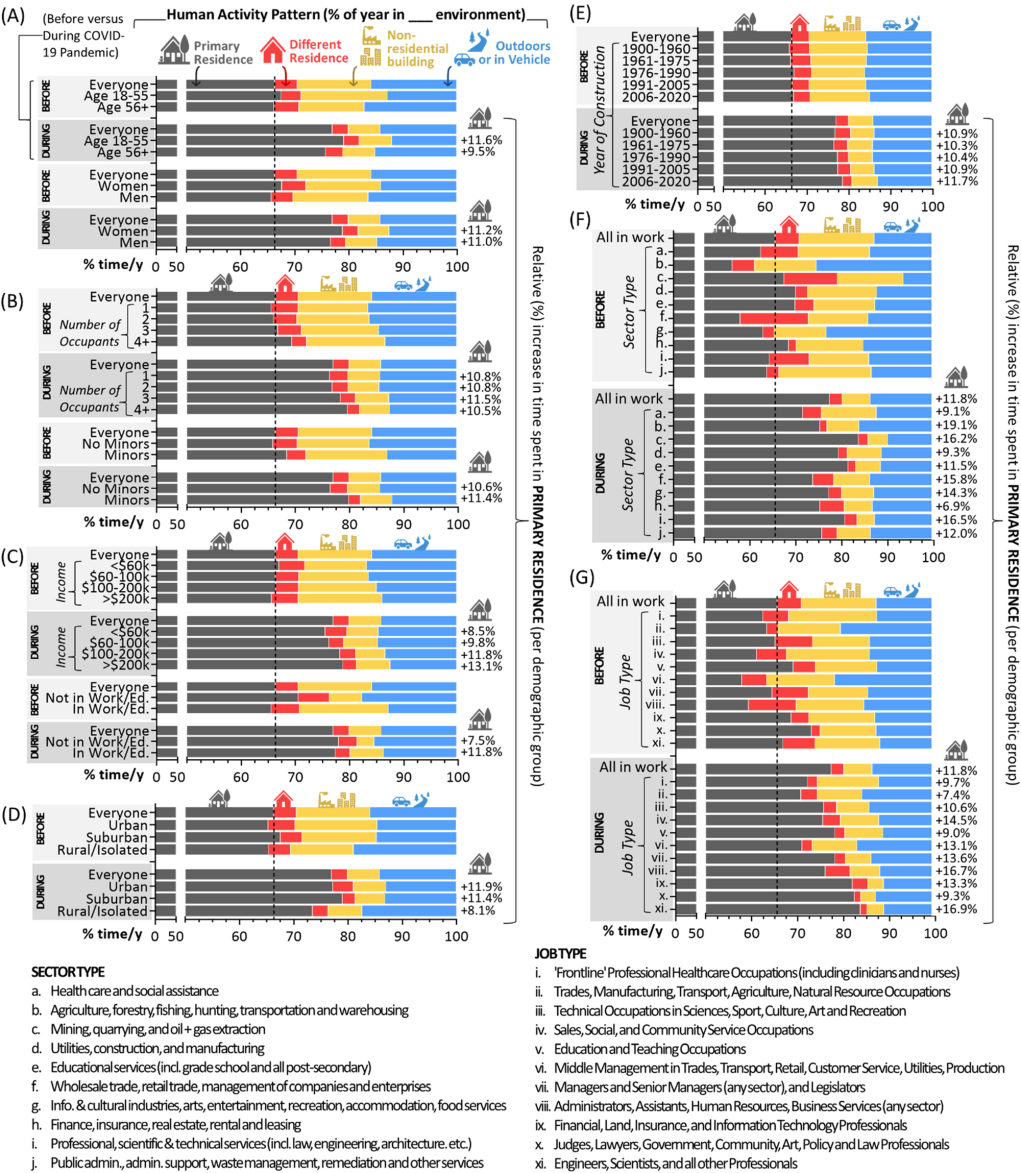
Activity patterns pre- and post-pandemic as a function of population demographics. In all cases, “everyone” refers to the total population for whom the specific demographic variable data was available. Panel **A**. Activity pattern by age and gender. Panel **B**. Household occupants and presence of minors. Panel **C.** Household income and work status of the primary respondent. Panel **D**. Primary residence community type. Panel **E**. Year of primary residence construction. Panel **F**. Sector of employment of the primary respondent, with legend shown below indicating sectors ‘a-j’. Panel **G**. Job of the primary respondent, with legend shown below showing jobs ‘i–xi’. Figures were prepared using Excel and GraphPad Prism 9.1.1 (225) (www.graphpad.com).

To distinguish workday from weekend activity patterns, those in work or education identified their number of working days per week as a function of winter (Dec, Jan, Feb), spring (Mar, Apr, May), summer (Jun, Jul, Aug) and fall (Sept, Oct, Nov) (Fig. 1I). The average number of workdays was ~ 4.5 per week, being highest in spring and fall, and lowest in summer. Participants then defined the number of hours per day spent in their primary residence, another residential building, a non-residential building (including offices, shopping centres, factories, recreation facilities, hotels, etc.), in a vehicle (of any type) or outside (including camping). For the pre-pandemic period, time spent in a non-residential building was greatest during workdays, while time spent in the primary residence was lowest (and time outside was highest) in summer (Fig. 2A). Using March 2020 as a reference point for disruptions caused by the arrival of the COVID-19 pandemic in Canada, we calculated activity patterns (% of year in a given environment) as a whole, and also as a function of seasons (Fig. 2B,C). A 10.6% increase (from 66.4 to 77%) in the amount of time people spent in the primary residence occurred after the COVID-19 pandemic began, mostly at the expense of time occupying a different residence or non-residential building. Overall time indoors did not change substantially, remaining at 84–86% of life, and comparable to previous observations^1^. For the population as a whole, 1062 additional hours per year were spent in the primary residence relative to the pre-pandemic period, with substantial variation between people (Fig. 2C,D). When examined as a function of demographics, we found that activity patterns as well as changes occurring after the COVID-19 pandemic differed by age but not gender (Fig. 3A), the number of occupants and whether there were minors present at home (Fig. 3B), household income and work status (Fig. 3C), community identity (Fig. 3D), year of primary residence construction (Fig. 3E), employment sector (Fig. 3F), and job type (Fig. 3G). The greatest increases in time spent in the primary residence per year after the onset of the COVID-19 pandemic were seen for those age 18–54, in work, with minors at home, in urban or suburban areas, in homes built after 2006, and in job types that encompassed professions more amenable to telecommuting, excluding frontline healthcare.

### Changes in radiation doses from residential radon gas after onset of the COVID-19 pandemic

**Figure 4:**
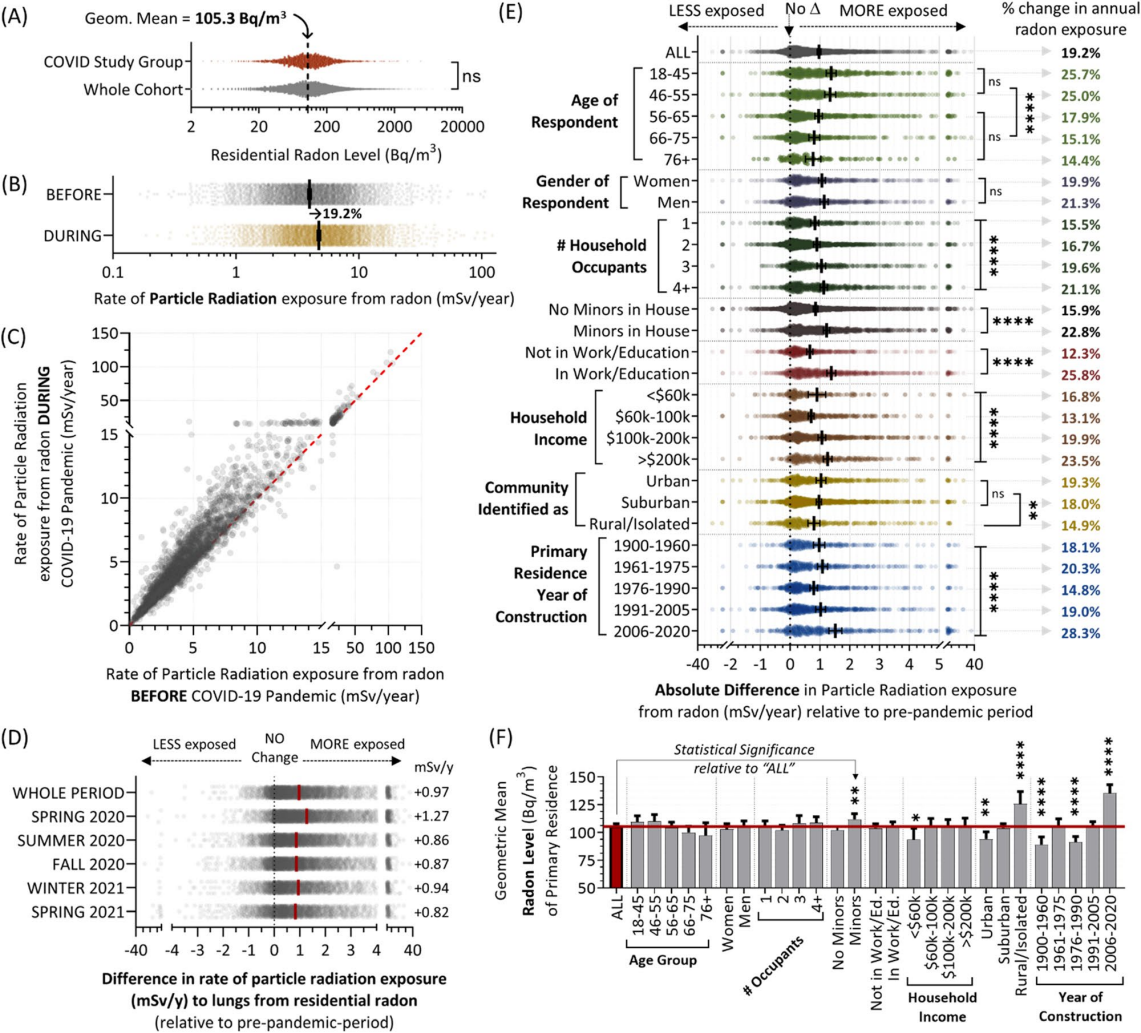
Annual dose rate of particle radiation (to the lungs) from residential radon gas inhalation before and after the onset of the COVID-19 pandemic. Panel **A**. Radon test outcomes for the activity pattern COVID-19 study group (red) relative to the larger *Evict Radon National Study* cohort (grey). Dashed line indicates the geometric mean of the study group. Panel **B**. Radiation dose rates (in mSv/y) for individuals the pre- (grey) and post- (yellow) pandemic period. Bars represent geometric means. Percent indicates the relative change in radiation dose rate to the lungs from residential radon for the post-pandemic period. Panel **C**. Scatter plot showing mSv/y radiation dose rate that individuals experienced from radon in their primary residence prior to (x-axis) or during (y-axis) the COVID-19 pandemic. Panel **D**. The data from (**C**) broken down by season. Panel **E**. The absolute difference in mSv/y radiation dose rate from residential radon being experienced by individuals relative to the pre-pandemic period, as a function of the indicated demographics. Percent values to the right indicate the relative % change in annual radon exposure. Panel **F**. The geometric mean radon level of the primary residence in Bq/m^3^ for all demographic groups indicated in (**E**). For all data, pairwise statistical comparisons are Mann–Whitney tests, while larger demographic group comparisons are 1-way ANOVA. **p < 0.01, ****p < 0.0001; ns = *p* > 0.05. Figures were prepared using Excel and GraphPad Prism 9.1.1 (225) (www.graphpad.com).

All households in our study population had completed (or were actively undertaking) long-term, 90 + day alpha track radon tests in their primary residence. Detailed radon test outcomes were described recently in^7,8^ and summarized in Fig. 4A. The geometric mean radon level of households in this study group was 105.3 Bq/m^3^, reflecting our larger radon test cohort and representative of Canadian averages^8^. By coupling this information with activity pattern data, and using a conversion formula standardized by the *International Commission for Radiological Protection* (see methods), we calculated annual dose rates of alpha particle radiation to the lungs (in mSv) from residential radon gas for all individuals for periods before and after the onset of the COVID-19 pandemic (Fig. 4B,C). There was a 19.2% relative increase in radiation dose rate for the population from radon in the primary residence, equating with an additional 0.97 mSv/y (Fig. 4D), representing a noticeably greater change than the 10.6% relative increase in time spent at home (Fig. 2). We speculated that this difference might be attributable to disproportionately higher radon exposure impacting people experiencing greater changes in their activity patterns. To explore this, we plotted the absolute difference in particle radiation dose rates relative to the pre-pandemic period for all previously described demographic variables (Fig. 4E) relative to these groups’ geometric mean residential radon levels (Fig. 4F). Increases in annual residential radon exposure were greatest for those who are younger, have children at home, work, have a higher household income, live in urban or suburban areas, and/or a newer primary residence. There were no significant (p > 0.05) or only very modest differences in the geometric mean radon levels as a function of age, household occupants, household income, or work status, suggesting that the primary driver of increased exposure in these groups was an altered activity pattern (more time at home), supported by our observations in Fig. 3. Greater exposures for those with minors at home, or who lived in newer residential properties corresponded with the phenomenon of newer North American properties containing the highest mean radon level^6,8^ (Fig. 4F), and being home to occupants who are younger, more likely to be working, and have children^7^ (Fig. 1H).

### Combinatorial trends linked to age, gender, employment, and having children living at home

**Figure 5:**
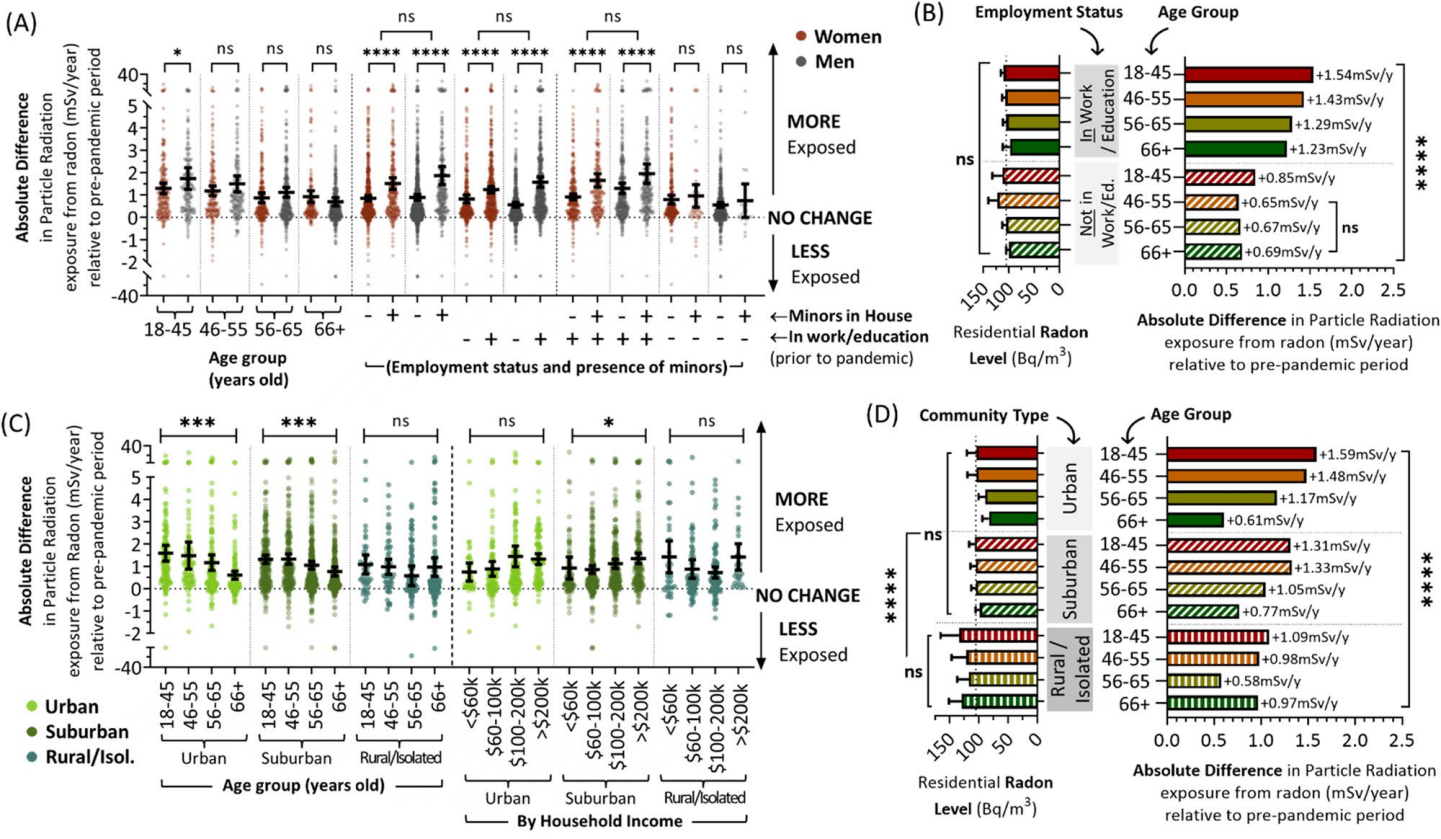
Increases in residential radon exposure after COVID-19 pandemic onset were greatest for those who are younger, have children at home, are in work, and/or live in urban or suburban areas. Panel **A**. The absolute difference in radiation dose rate (mSv/y) from residential radon relative to the pre-pandemic period being experienced by women (red) and men (grey), as a function of age, employment status and the presence of minors. Panel **B**. Combining employment status and age groups (from A), the absolute difference in radiation dose rate from radon (mSv/y), relative to residential radon level (Bq/m^3^). Panel **C**. The absolute difference in radiation dose rate (mSv/y) from residential radon relative to the pre-pandemic period being experienced by people living in urban (light green), suburban (dark green), or rural/isolate areas (dark cyan) as a function of age or household income. Panel **D**. Combining community type and age groups (from **C**), the absolute difference in radiation dose rate from radon (mSv/y), relative to residential radon level (Bq/m^3^). For all data, pairwise statistical comparisons are Mann–Whitney tests, while larger demographic group comparisons are 1-way ANOVA. *p < 0.05, ***p < 0.001, ****p < 0.0001; ns = *p* > 0.05. Figures were prepared using Excel and GraphPad Prism 9.1.1 (225) (www.graphpad.com).

We observed no significant (*p* > 0.05) differences in annual radiation dose rates from residential radon between those identifying as women or men (Fig. 4E), reflecting the lack of difference in either their household radon levels (Fig. 4F) or activity patterns (Fig. 3A). To explore gender effects more closely, we distinguished women from men based on age, the presence or absence of minors in the home, and/or employment status. With the exception of a small but significant (p < 0.05) difference between men and women aged 18–45, wherein younger men experienced a larger increase in radon exposure after the pandemic relative to women, there were no other significant (p > 0.05) differences as a function of gender for all other demographic combinations (Fig. 5A). We also noted that the impact of having minors living in the primary residence (or visiting regularly) was negated (p > 0.05) when combined with employment status, as the overwhelming majority of those with children ‘at home’ were also working. Age and employment status, on the other hand, had independent impacts when examined in combination, with decreased age and being in work both significantly (p < 0.0001) contributing to higher radiation dose rates (Fig. 5B, right graph). These effects were independent of household radon level (Fig. 5B, left graph), indicating they are driven by activity pattern changes (Fig. 3A,C).

### Combinatorial trends linked to age, household income, and community type

Our analysis of post-pandemic onset changes in residential radon exposure (Fig. 4E) revealed significant (p < 0.01) differences between community types, with those in urban or suburban areas experiencing the greatest increases in annual radiation dose rates versus those in rural or isolated communities. This difference was notable as rural area properties contain significantly (p < 0.0001) and substantially greater household radon levels relative to urban or suburban equivalents, and emphasizes the observation that the activity patterns of people living in rural areas changed the least after the COVID-19 pandemic, relative to any other community type (Fig. 3D). To explore community-related trends more closely, we separated them on the basis of age, and household income (Fig. 5C). Of note, age-related trends on increased radon exposure were not significant (p > 0.05) in rural/isolated communities, whilst being substantial and significant (p < 0.001) for people living in both urban and suburban communities. The significance of household income was largely erased (p > 0.05) when examined by community type, with only a modest effect in suburban regions remaining significant (p < 0.05). Overall, the largest increase in radiation dose rate from residential radon after the pandemic was seen in urban populations aged 18–55, with the smallest increases experienced by those aged 56–65 in rural areas (Fig. 5D).

### The impact of employment sector and job type on post-pandemic radon exposure

**Figure 6:**
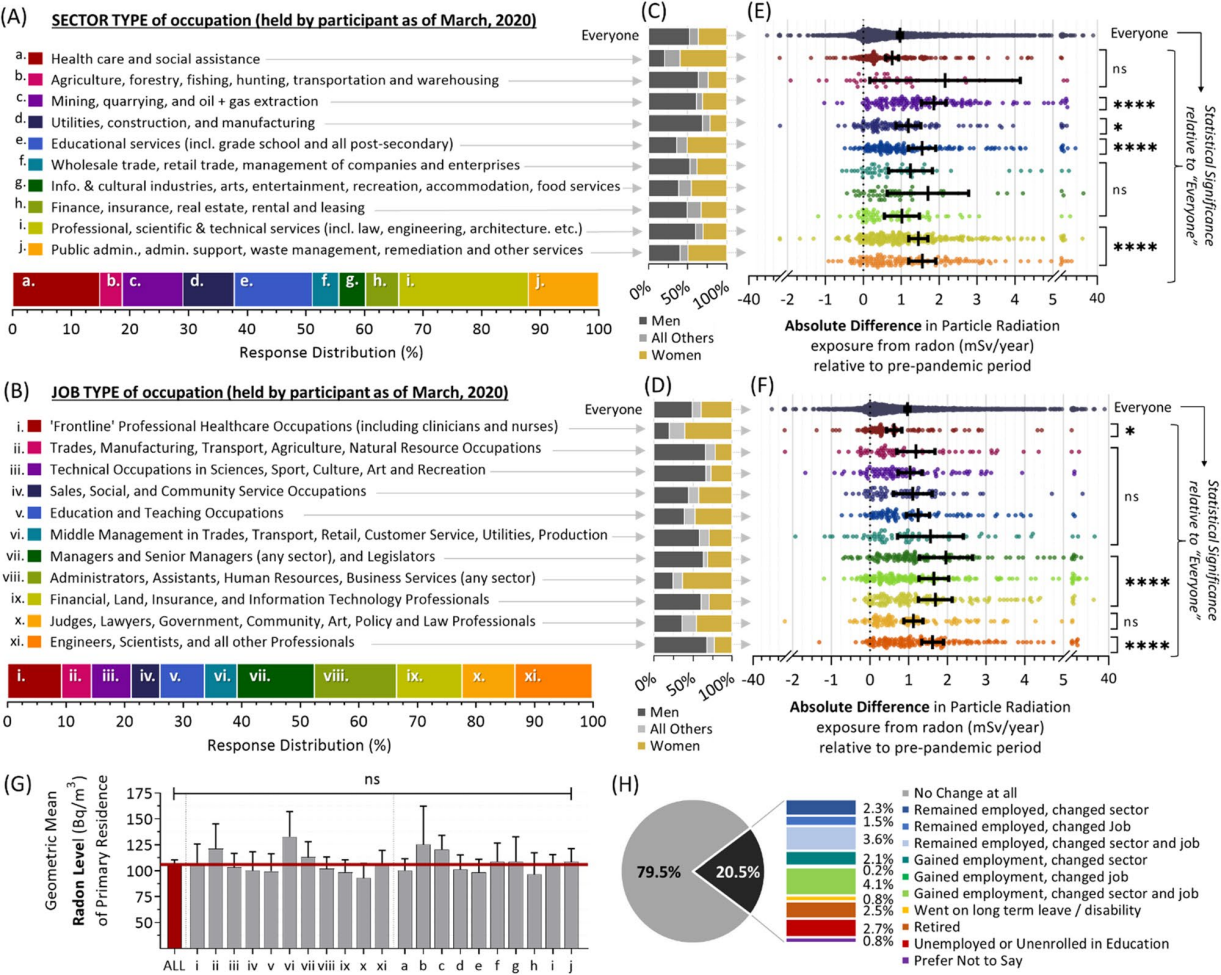
Increases in residential radon exposure after COVID-19 pandemic onset as a function of sector and job type. Panel **A**. Relative distributions of sectors of employment held by participants at the start of the COVID-19 pandemic in Canada (March 2020). Panel **B**. Relative distributions of job types performed by participants at the start of the pandemic. Panels **C**, **D**. Gender distributions associated with indicated sector and job types from (**A**, **B**). Please note, in this case, “all others” refers to the combination of gender minorities together with all those who did not report their gender identity. Panels **E**, **F**. The absolute difference in radiation dose rate (mSv/y) from residential radon relative to the pre-pandemic period as a function of sector (**E**) and job (**F**). Panel **G**. The geometric mean radon level of the primary residence in Bq/m^3^ for all demographic groups indicated in (**E**, **F**). Panel **H**. Distribution of those experiencing a change in employment (or not) following the onset of the COVID-19 pandemic. For all data, pairwise statistical comparisons are Mann–Whitney tests, while larger demographic group comparisons are 1-way ANOVA. *p < 0.05, ***p < 0.001, ****p < 0.0001; ns =*p* > 0.05. Figures were prepared using Excel and GraphPad Prism 9.1.1 (225) (www.graphpad.com).

As both employment sector and job type were associated with substantial differences in activity pattern changes following the onset of the COVID-19 pandemic (Fig. 3F,G), we next ascertained whether these variables equally impacted residential radon exposure. It is worth noting that different sectors and/or job types also have distinct gender profiles, most of which are not balanced (Fig. 6A-D). For example, while health care and social assistance sectors are over-represented by women, those in mining, quarrying and resource extraction are more likely to be men (Fig. 6C). Similarly, those in roles as administrators, assistants or human resource professionals are more likely to be women, while people working jobs in the trades, manufacturing, transport, and agriculture are more likely to be men (Fig. 6D). With this in mind, we found that there were significant (p < 0.05 to p < 0.0001) differences across both sectors and jobs (Fig. 6E,F), with the most substantial increases in residential radon exposure linked to managerial and administrative positions, or professional job types excluding healthcare; this is likely driven by the relative feasibility of doing these types of jobs via a work-from-home arrangement. Of note, there were no statistically significant differences (p > 0.05) in household radon level as a function of job or sector (Fig. 6G), indicating that differences are driven by the marked activity patterns variations by job and sector (Fig. 3F,G). It is important to note that we controlled for employment changes following COVID-19 pandemic onset. While a majority (79.5%) reported no change, 20.5% experienced a shift in their work status encompassing retirement, changes in job and/or sector, leave (of any type), or a loss or gain of employment (Fig. 6H). Hence, a minority of activity pattern shifts (following the pandemic) are likely to be driven by employment changes.

### Pandemic-driven radon exposure differences: decreases for some, minimal changes for others, and largest increases for younger age groups

**Figure 7:**
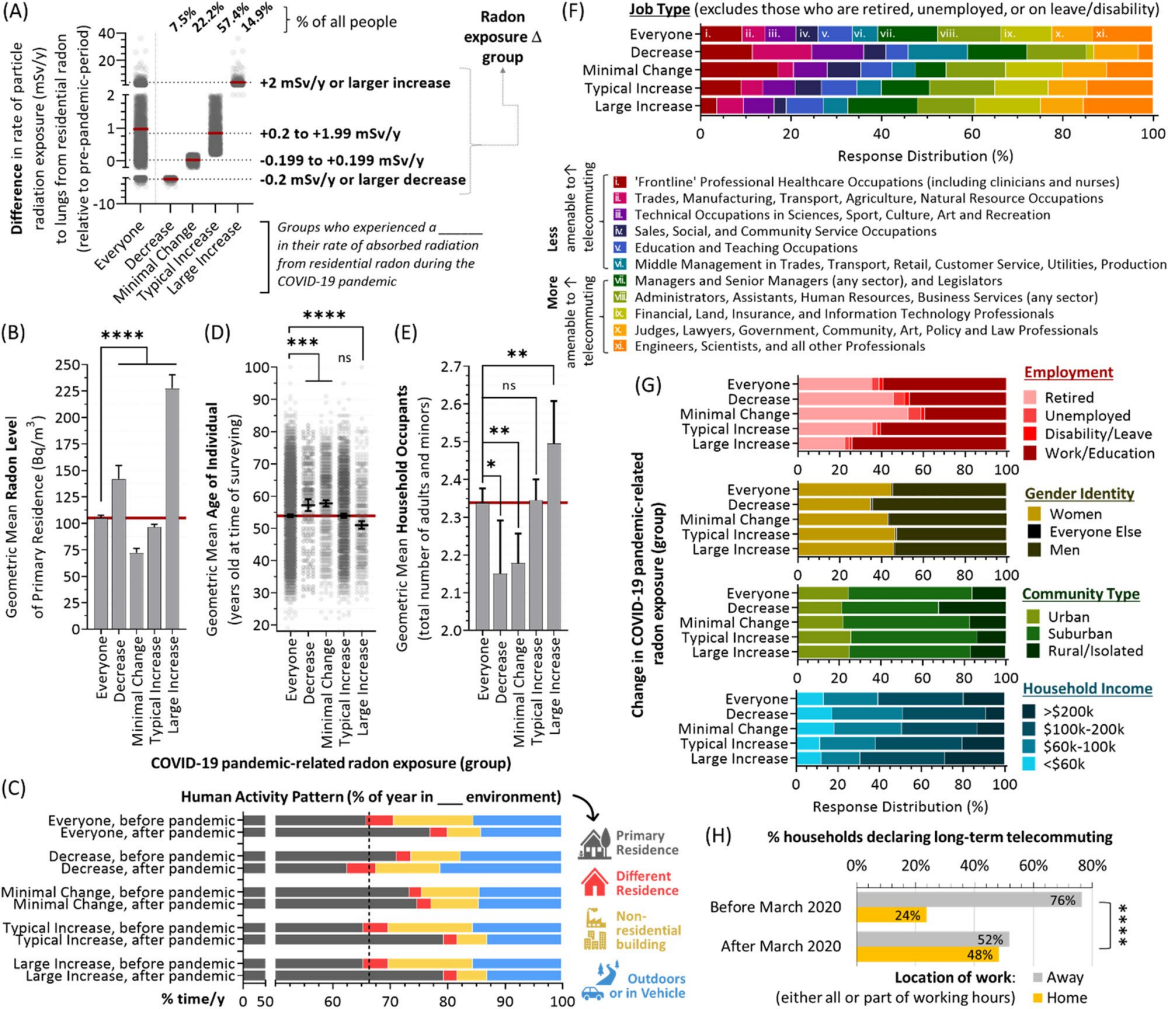
Those experiencing exceptionally large increases in radon exposure following the COVID-19 pandemic are younger, in homes with more occupants, and in telecommuting-amenable jobs. Panel **A**. The absolute difference in radiation dose rate (mSv/y) from residential radon relative to the pre-pandemic period broken down into the four indicated groups. Panel **B**. The geometric mean radon level of properties occupied by those in COVID-19 pandemic-related exposure groups defined in (**A**). Panel **C**. The before and after COVID-19 pandemic onset activity patterns for those in groups defined in (**A**). Panel **D**. The geometric mean ranges of those in COVID-19 pandemic-related exposure groups defined in (**A**). Panel **E**. The geometric mean number of household occupants for the COVID-19 pandemic-related exposure groups defined in (**A**). Panel **F**. Distribution of job types held by those in COVID-19 pandemic-related exposure groups defined in (**A**). Panel **G**. Distribution of employment status (reds), gender identity (yellows), community type (greens), and household incomes (blues) for those in COVID-19 pandemic-related exposure groups defined in (**A**). Panel **H**. The percent of households declaring that one or more occupants telecommute (work from home) either all or part of working hours, before and after March of 2020. Pairwise statistical comparisons are Mann–Whitney t-tests. *p < 0.05, **p < 0.01, ***p < 0.001, ****p < 0.0001; ns = *p* > 0.05. Figures were prepared using Excel and GraphPad Prism 9.1.1 (225) (www.graphpad.com).

An examination of individual-level activity pattern and radon exposure data in Figs. 2D and 4C reveals that, although Canadians collectively experienced greater radiation doses from radon due to increased time in the primary residence during the pandemic, there are some who saw little to no change or even a decrease. To study this in greater depth, we separated the study population into four groups based on their change in radiation exposure (mSv/y) from radon relative to pre-pandemic period (Fig. 7A). Relative to the (57.4%) majority who experienced a ~ 0.2–2 mSv/y increase, a sizeable percentage (22.2%) saw less than a 0.2 mSv/y (i.e., minimal) change in their radiation exposure. A further 7.5% saw a notable decrease in radon exposure, while 14.9% are experiencing a very substantial increase of up to 40 mSv/y. Paradoxically, those who experienced a substantial decrease in their annual radon exposure were in properties with higher household radon levels compared to the cohort as a whole (Fig. 7B). This contradiction is explained by differences in activity pattern changes, with those in the decreased exposure group representing people whose time in the primary residence decreased after the pandemic, offsetting their comparatively greater property radon levels (Fig. 7C). This decreased radon exposure group was significantly (p < 0.001) older, lived in homes with fewer occupants, were more likely to be employed in sectors or jobs less amenable to telecommuting or not working at all, have lower household incomes, and/or who lived in rural-identifying communities (Fig. 7D-G).

Activity patterns also explain the difference between those who saw minimal change in radon exposure (i.e., their time spent at home did not appreciably change after the pandemic) from those experiencing any sort of an increase in radon exposure (whose time at home increased substantially) (Fig. 7C). In terms of demographics, people who experienced only a minimal change in post-pandemic radon exposure were older than the majority, had fewer household occupants, and were either more likely to not be working or were employed in professions such as frontline healthcare, trades, transport, agriculture, or technical roles that are less amenable to remote work (Fig. 7D–G). There was no difference in activity pattern changes between the ‘typical’ and large radon exposure increase groups and, in this case, differences are driven by drastically higher property radon levels for those in the large increase group (Fig. 7B,C). These individuals (experiencing the greatest increase in radon exposure) were also significantly (p < 0.0001) younger, lived in households with more occupants, and were more likely to be working and in managerial, administrative or professional roles understood to be more amenable to post-pandemic telecommuting (Fig. 7D–G)^23,24^. By comparing individuals reporting intentions before/after March 2020, we observed a doubling (24% rising to 48%) in long term telecommuting (Fig. 7H), suggesting that the trends we observed may underlie a new status quo^37,38^.

### An intervention to encourage health-seeking behaviour amongst the most impacted groups

A new status quo of a (further^7^) heightening in radon exposure amongst younger people is a troubling prospect. To counter this, we trialled a behavioural intervention to determine whether we could improve radon awareness and testing uptake in adults aged 20 to 45. As we had already observed that younger people were more likely to become aware of radon via word of mouth or social media^29^, we explored whether communication delivered by ‘microinfluencers’ might be an effective tool to encourage health-seeking behaviour (radon testing) amongst younger adults. Microinfluencers are non-traditional, often local celebrities with between 5000 and 50,000 social media subscribers that demonstrate high degrees of trust (in the influencer) via parasocial interactions and relationships^48^. Microinfluencer marketing has been found to be effective in the context of tobacco reduction for younger people^49^ and, most recently, vaccine hesitancy among under-served USA populations^50^, but systematic evaluation in the context of public health messaging remains limited. Ten Instagram microinfluencers located in the Canadian province of Alberta were hired in spring and summer of 2021 to deliver at least three curated posts describing radon health effects, what radon testing involved, and how to interpret test outcomes for their own properties (see Supplemental Information, Section II). We considered that people who subscribed to at least one microinfluencer would receive a minimum of 3 interactions with radon information during our study. This was by design, as we had determined previously that 65% of people require 2–5 interactions with radon information before obtaining a test within a several month period^29^. By the end of the test period, microinfluencers delivered an average of 3.2 posts each and, combined with ‘Instagram Stories’ (ephemeral content available only for 24 h), posted a total of 64 unique pieces of radon awareness and testing-related content to subscribers.

**Figure 8:**
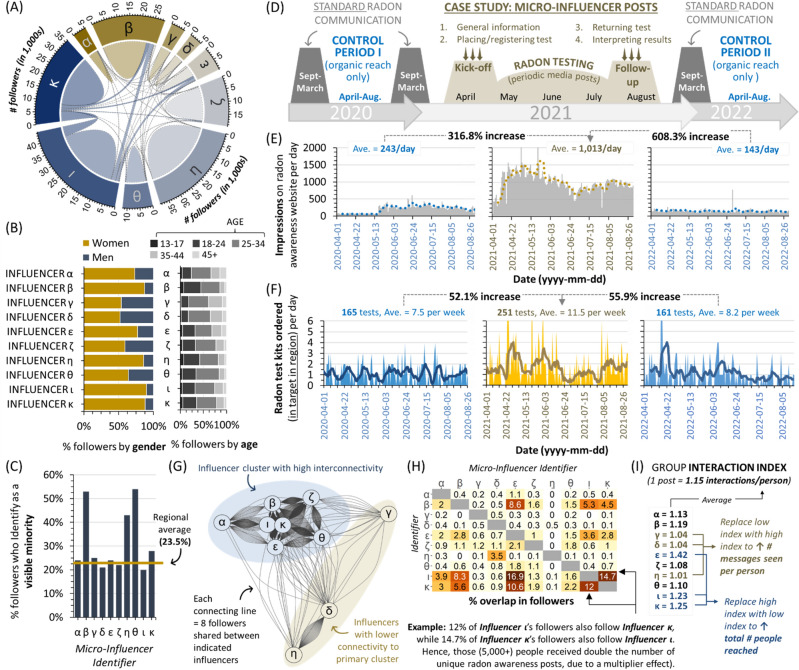
Microinfluencer based public health communication is effective at increasing health-seeking behaviour (radon-testing) amongst younger adults. Panel **A**. A chord diagram indicating the number of followers (in the 1000 s) per influencer designated by the indicated Greek letter identifiers, with lines between influencers indicating shared followers. Panel **B**. Per influencer from (**A**), the percent distribution of followers by age and gender identity. Panel **C**. Per influencer from (**A**), the percent of followers identifying as a visible minority. Pane **D**. Schematic of activity over the case study and control periods. Panel **E**. Radon awareness study website impressions per day across the control and case study periods. Panel **F**. Radon test kits obtained per day from the study and only from the target region, across the control and case study periods. Panel **G**. A network diagram showing relative interconnectivity between influencers. Each line connecting an influencer represents 8 total shared followers. Panel **H**. Matrix diagram showing the percent of shared followers for all pairwise combinations. Panel **I**. The overall interaction index (i.e., the number of interactions with radon awareness information received per follower, per social media post) for the group, based on each individual. Figures were prepared using Excel, R Studio and GraphPad Prism 9.1.1 (225) (www.graphpad.com).

For analysis, microinfluencers were assigned Greek letter identifiers (alpha to kappa), with the number of people subscribing to their social media channel (‘followers’) indicated in the chord diagram in Fig. 8A. Microinfluencers were included on the basis of their followers being predominantly aged 25–44. Subscribers were over-represented by women, but otherwise reflected (or exceeded) regional visible minority averages (Fig. 8B,C). The case study period was April to August 2021, and was compared to identical control periods in the year before and after, during which no online awareness activities were carried out in the region by our team (i.e., only organic reach) (Fig. 8D). Standard periods of radon awareness activity (including paid ads on social media) were from September to March of a given year, and were excluded to reduce confounding variables. During the test period, followers were encouraged by microinfluencers to visit the radon study website for further information, and to obtain a radon test for their own properties. To monitor outcomes, we measured impressions and new users at (i.e., visits to) the study website (Fig. 8E), as well as the number of radon study test kits ordered per day **(**Fig. 8F). A marked increase in interactions with radon awareness information (website impressions increased 316–608%) and health-seeking behaviour (regional households obtaining a radon test increased 52–56%) was observed during the case study, relative to control periods.

Finally, we retrospectively analyzed how many people subscribed to multiple microinfluencers (i.e., follower inter-connectivity), in order to determine how future behavioural interventions of this type might be enhanced. Network analysis revealed that there was a cluster of influencers (indicated in blue) with high follower interconnectivity, meaning that their subscribers were more likely to follow > 1 influencer within the cluster. This group was markedly distinct from microinfluencers gamma, delta, and eta (indicated in yellow), whose subscribers were much less likely to follow microinfluencers in the main (blue) cluster (Fig. 8G). Using a matrix, we then visualized the % shared followers between all possible pairings, which ranged 0–16.9% (Fig. 8H). This data was then used to calculate an ‘interaction index’ (see methods, and Supplemental Information, Section III) for the microinfluencer group overall, reflecting the average number of public health information encounters per person that was generated by a microinfluencer posting one message on their channel. In this context, any interaction index exceeding 1 implies that a fraction of subscribers will receive the public health information ≥ 2 times from separate microinfluencers, and is essentially a numerical measure of amplified ‘word of mouth’. For this cohort of microinfluencers, each social media post resulted in 1.15 radon awareness interactions per person (Fig. 8I). With an average of 3.2 posts per influencer, we can infer from this interaction index that subscribers encountered radon awareness information at least 3.7 times over the study period, and potentially as high as 7.4 times if they additionally viewed transient Instagram Stories.

## Discussion

This study represents an update of activity patterns for both the immediate pre- and intra-COVID-19 pandemic periods. Relative to the 2001 Canadian and American *National Human Activity Pattern Study,* which found adults of that time spent 86.9% of life indoors and 68.9% (6018 h/y) inside the home^1^, the activity patterns we observed for the immediate pre-pandemic period (2018–2019) were comparable, at ~ 84% of life indoors and 66.4% (5815 h/y) spent at inside the primary residence. A key takeaway message from this work, however, is that the onset of the COVID-19 pandemic marks a sudden and widespread alteration in human activity patterns, a behavioural determinant of health. While most people are not spending any more or less time indoors (still ~ 86% of life, post March 2020), there have been average increases of > 1000 h in the amount of time spent at home in a year (equating to 77% of life at home), and this remained consistent beyond the periods of enforced lockdowns that were part of the initial COVID-19 pandemic response in the study region. While we do not suggest that the ‘mid-pandemic’ magnitude in activity pattern shifts observed from 2020 to 2021 will be permanent, we speculate that they will not return to the pre-pandemic baseline of 66–69% of life at home, and will likely stabilize over the long term at an intermediate value (relative to the early pandemic extremes) due to the normalization and demand for hybrid work arrangements across sectors.

Changing activity patterns mean changing health risks driven by indoor environmental toxicants such as residential radon. This collateral consequence of the COVID-19 pandemic is widespread but not universal, and we find differs by age, work status, job type, employment sector, community type, and other demographic variables. Those who are younger, in work, and who live in urban and suburban regions are among the most impacted, and are now experiencing significantly increased doses of radiation to their lungs from residential radon gas exposure. Based on all available information regarding radon exposure^2,9–11,13,14,16,20^, this is expected to increase lifetime lung cancer risk for populations moving forward. Additionally, we note that the workers who are most impacted (i.e., those working in office/desk jobs that are now more likely to be performed from a residential property for at least part of the time) are a group that is not traditionally considered in occupational health campaigns. This marks a sudden but important shift in how those with responsibility for workers’ health will need to consider cancer prevention programming in the future.

Global, pre-pandemic average annual radiation doses from radon are an estimated 1.2 mSv/y, with some North American populations absorbing far higher annual doses^5–8,15,21,24,25^. Indeed, before the pandemic, average Canadian radiation exposures from radon were ~ 4 mSv/y^7,26^. Our work shows this rose by ~ 1 mSv/y after March of 2020, so that intra-pandemic averages reached ~ 5 mSv/y (Fig. 4). For those aged 18–45, average increases in radiation dose from radon gas were much higher, at + 1.5 mSv/y (Fig. 5B), and as high as + 40 mSv/y in extreme cases (Fig. 7A). If even a proportion of this increase is sustained over the long term, as is predicted from the new status quo of telecommuting behaviour (supported by our findings in Fig. 7H, and the work of others^37,38^), then we predict near-future increases in lung cancer risk in the general population unless this is countered by increased radon exposure reduction strategies and/or individual behaviours. Hence, from a policy perspective, a key recommendation that arises from this study is to encourage regulators and employers with full or hybrid telecommuting workforces to consider radon as an occupational carcinogen warranting legislatively required controls. Indeed, many jurisdictions have a legally enforceable ‘general duty clause’ that requires employers to ensure a work environment does not carry undue risk of injury or occupational disease to any person^51^; this is especially true in the case of ionizing radiation sources for a wide variety of work environments, including radioactive sources of natural origin (such as radon)^40,44,52–54^.

It is important to note that long term inhalation of 100 Bq/m^3^ radon in indoor air is the minimum exposure that incurs a statistically significant (16%) increase in lifetime risk of lung cancer and, as discussed earlier, equates to an estimated 4 mSv/y annual radon exposure^2,7,14,15,24,25^. Before the pandemic, 50.8% of Canadians experienced annual doses ≥ 4 mSv/y from residential radon gas^7,26^, and our work here shows that this proportion approached 60.4% during the COVID-19 pandemic. This emphasizes a shift in baseline radon gas exposures for a great number of people into the cancer-causing range, and especially so for younger adults. It is important to emphasize that Canada already displays elevated rates of lung cancer that are independent of tobacco smoking behaviour, with Canadian lung cancers being (for example) 163% greater than Sweden, despite both having ~ 12% smoking rates, similar climates, comparable population demographics, but very different modern residential radon levels, being 467% higher in a new Canadian home versus a Swedish equivalent (discussed in detail in^8^).

In terms of study strengths and weaknesses, we acknowledge that we have not considered the issue of ethnicity within this work, nor did we obtain sufficient data on sexual or gender minorities to measure differences in either activity pattern or radon exposure relative to other groups. People of racial, sexual, and gender minorities experience structural marginalization, resulting in distinct social and structural determinants of health that are important to assess. We were, however, able to calculate activity patterns of those experiencing disability, and surveyed populations of lower household income levels linked to marginalization, as well as disparities in radon awareness, exposure, and/or lung cancer risks^26,55,56^. We also note that there are some apparent contradictions in increased radon exposure between employment sectors and closely related jobs; for example, the mining, quarrying and oil and gas extraction sector (who had significant collective increases in radon exposure, p < 0.0001) versus those performing jobs in natural resource extraction (non-significant change, p > 0.05). In this case, the confounding effect is caused by grouping managerial and administrative jobs together with frontline workers of the same sector. Thus, we suggest that an examination of an individual or group’s specific role (job) within a given sector is a more accurate indicator of their activity pattern, and this reflects common knowledge in occupational epidemiology (i.e., that job title is a more specific indicator of exposure than industry/sector)^57^. A key strength of this work is that we were able to determine differences on the basis of urban versus rural identity, which is often over-looked in studies of this type, as well as integrate information from the built environment (i.e., property ages) with human demographics to capture a more holistic overview of how the COVID-19 pandemic has impacted activity patterns. We were also able to analyse outcomes based on individualized radon exposures and activity patterns, and did not rely on any regionalized or aggregate assumptions regarding either in source data.

On another positive note, our work adds to the growing evidence^49,50^ that that microinfluencer-based public health messaging is an impactful intervention for enabling health seeking behaviour, in this case amongst younger people most impacted by altered radon exposure due to shifted activity patterns. Our microinfluencer case study observed large gains in radon awareness and testing achieved using this technique, and we developed tools (i.e., the interaction index) that may prove useful by public health communicators seeking to motivate behaviour change in younger people. For a given microinfluencer group, we suggest that higher interaction indices may be preferable to promote behaviour change, as it is more likely that a person is encouraged towards a call to action when they hear the message from multiple people and/or more than once (Supplemental Information, Section III, and Fig. 8I). Conversely, a lower interaction index might be desired to optimize the overall reach of information dispersal, where more people in total will see a single message at least once from at least one influencer. We suggest that a key next step is to determine whether this approach is scalable, applies across a diversity of regions or demographic groups, and if it is also efficacious at addressing other public health concerns.

In summary, our work supports the re-evaluation of environmental health risks modified by changing activity patterns and presents a clear case for one widespread collateral consequence of the COVID-19 pandemic being elevated exposure to radioactive radon gas inhaled within the indoor air of the residential built environment.

## Methods

### Statement of approvals

All activities were pre-approved by the Conjoint Health Research Ethics Board, Research Services, University of Calgary (IDs = REB17-2239, REB19-1522) or the Health Research Ethics Board of Alberta, Cancer Committee (HREBA.CC-17-0246), adhering to citizen science research best practice^58^, and in accordance with all regional guidelines and regulations. This work peer-reviewed in advance and funded by three public sector agencies: Health Canada, the Canadian Institutes for Health Research, and the Alberta Real Estate Foundation. The research team was head-quartered at the University of Calgary and University of British Columbia, and involved the staff of the *Evict Radon National Study*, CAREX (Carcinogen Exposure) Canada.

### Enrollment and surveying

All participants active within the *Evict Radon National Study* as of 2021 were invited to complete online surveys and questionnaires using the Qualtrics survey platform. Study enrollment for this study was based on convenience recruitment for all wanting to join, with all adult homeowners and renters in any residential building type being eligible. Enrollment opportunities were communicated broadly, and across the study region via multiple platforms including online and mass media via professional journalism. No data from any constituent part of this cohort were from known or pre-selected lung cancer cases. Wherever possible, data was expressed as a function of single or combined demographics to contextualize outcomes. In addition to the detailed analyses presented in this study, many of the human demographics and building metrics associated of this cohort have been previously peer-reviewed and described in^6–8,26,29^. Records of informed consent were obtained in all cases, and participants were permitted to withdraw at any time. All identifying information was removed early on during data analysis, and absolutely no identifying information is included within the data presented in this study for publication. A complete list of survey questions is outlined in Supplemental Information, Section I.

### Microinfluencer case study

From March to August of 2021, ten microinfluencers (people with ~ 5000–50,000 followers) on the Instagram social media platform (owned by Meta) that were based in the Canadian province of Alberta (i.e., the ‘case study region’) were hired to post > 3 curated radon awareness pieces on their primary channels. Influencers were selected by Glacier Communications Inc. (headquartered in Calgary, Canada), with inclusion criteria based on being located within the case study region, having followers that were primarily between 20 and 44 years of age and identified predominantly as women. Knowing demographics such as age and gender of a given subscriber group in advance was possible as Meta provides microinfluencers basic, aggregate, de-identified demographic metrics of their followers as part of a professional dashboard. In the study year (2021), total subscribers for this microinfluencer cohort equalled 202,072 accounts (caveat: not all accounts might have been active, some might be operated by > 1 person, and there exists the potential for some being automated ‘bots’). Curated Instagram social media posts that were conceptualized, produced, and delivered by microinfluencers cost an average of CAD$284 each, and focused on the nature of radon health impacts, obtaining and placing a radon test, receiving, and interpreting the results. Posts were delivered to subscribers over the spring to summer period, during which a majority of national and regional radon awareness information programs were on hiatus. Some microinfluencers additionally chose to deliver Instagram Stories (which last 24 h) coupled with their main posts, and this was considered value-added material. Over the course of the case study, impressions, and new users on the Evict Radon National Study website (EvictRadon.org) were monitored using Datorama and Google analytics, while the number of study test kits ordered was ascertained from the Shopify web platform (www.shopify.ca). The two ‘negative’ control periods included the equivalent periods in 2020 and 2022 during which no microinfluencer (or other confounding communication) activity from the *Evict Radon National Study* within the case study region took place. A sample of publicly available microinfluencer posts (featuring, produced, and posted by the microinfluencers hired as part of this work) from the period of activity are shown in Supplemental Information, Section II.

### Radon testing and radiation exposure calculations

From 2015 to 2021, Canadians purchased RadTrak2 or Radtrak3 alpha track 90 + day radon detectors that they then deployed, returned for analysis, and later received their specific radon reading in a confidential manner. Radon outcomes for this cohort were reported recently in^7^. Non-profit study kits ranged from CAD$45–53, depending on year (price differences driven by inflation and material costs across 2015–2021). To convert Bq/m^3^ indoor air radon levels to human mSv radiation exposures (to lungs), the ICRP provides the following formula^59^:

*(6.7×10*^*-6*^* mSv per Bq·h/m*^*3*^*) × ([radon] Bq/m*^*3*^*) × ([time in residence per year] h/y) = mSv/y*.

Values for the amount of time spent in the primary residence per year for a typical adult (*“time in residence per year”)* were calculated from individually reported residential occupancy data from 1128 participants, and cross-referenced with data from the *National Human Activity Pattern Study* (NHAPS)^1^. The NHAPS, which included responses from both Canadian and American respondents, estimated that 68.7% of life was spent inside a residence for the average adult. Of the total 8760 h per year, this equates to 6018 h/y and represents an average of all different employment statuses. Participants reported their data by season (Winter, Spring, Summer, Fall), with weekend/holiday versus workdays accounted for within the questionnaires described in detail in^7^. All response-derived “*time in residence per year”* outcomes were linked to individually reported employment statuses and used to extrapolate the same values for all remaining participants (2390) for which employment data was collected. Please note that for the purposes of participant responses in this survey, being enrolled in full or part time formal education (at a university, college, technical school, etc.) was considered to be a type of employment and grouped with those responses.

### Calculating interaction index for microinfluencer groups

Data were analyzed using Microsoft Excel and R (Version 4.2.1). Instagram followers were captured June 2022, using the Google Chrome (Version 103.0.5060.134) extension “InsFo—Export Instagram Followers to CSV” (Version 3.0.7). The chord diagram in Fig. 8 1 was generated using the R package “circlize”^60^. The number of shared followers for each influencer were determined pairwise for all influencers. Using these values, the interaction index was determined by comparing the summed total of all shared followers to the total number of followers *per* influencer. For example, to determine the interaction index for influencer α, the following formula was used:

$${interaction\, index}_{\alpha }= \frac{\sum_{\alpha }^{\kappa }Shared\, followers}{{Total\, followers}_{\alpha }}$$

The resulting interaction indices were then averaged to calculate the overall interaction index of the entire influencer cohort. For reference, an interaction index of 1 means that there are no shared followers, while values > 1 indicate the average number of times a follower is exposed to the desired messaging. The analysis R code is available upon request.

### Statistical analysis

Statistical analyses were carried out using Excel and GraphPad Prism 9.1.1 (225) (www.graphpad.com). One-way ANOVAs were carried out to test differences between groups (e.g., year of construction, occupant age, mSv, etc.), with Bonferroni-Holm corrected post-hoc testing carried out to characterize group differences for pairwise comparisons if the ANOVA reached significance. Mann–Whitney pairwise nonparametric t-tests were used to assess the significance of scatter plot data.

## Supplementary Information

Supplementary Information.

## Data Availability

The de-identified raw data sets generated by the current study are available to researchers following reasonable requests to Dr. Goodarzi, and will require a legally binding data transfer agreement. Data may not be used for private, commercial, or for-profit purposes for any reason.
