# Supplementary material for: Consequences of changing Canadian activity patterns since the COVID-19 pandemic include increased residential radon gas exposure for younger people
Source: Sci Rep. 2023 Apr 7;13:5735. doi: 10.1038/s41598-023-32416-8 (PMC10081328; doi:10.1038/s41598-023-32416-8)
Supplement: Supplementary file 1 — Supplementary Information. [file 41598_2023_32416_MOESM1_ESM.pdf]

SUPPLEMENTAL FILE

for

Changing North American activity patterns since the COVID-19 pandemic have increased residential radon gas exposure, especially for younger people

Natasha L. Cholowsky[1*], Myra J. Chen[1*], Ghozllane Selouani[1], Sophie C. Pett[1], Dustin D. Pearson[1], John M. Danforth[1], Shelby Fenton[2], Ela Rydz[2], Matthew J. Diteljan[3], Cheryl. E. Peters[2,4**], Aaron A. Goodarzi[1,2**]

Author Affiliations:
[1]Robson DNA Science Centre, Department of Biochemistry & Molecular Biology, Charbonneau Cancer Institute, Cumming School of Medicine, University of Calgary, Alberta, Canada; [2]Department of Oncology, Charbonneau Cancer Institute, Cumming School of Medicine, University of Calgary, Alberta, Canada; [3]Glacier Communications, Inc., Calgary, Alberta, Canada; [4]British Columbia Centre for Disease Control, British Columbia Cancer, School of Population and Public Health, University of British Columbia, British Columbia, Canada.
*These authors contributed equally.
**Co-Corresponding authors are: C. Peters (cheryl.peters1@bccdc.ca) and A. Goodarzi (a.goodarzi@ucalgary.ca)

SECTION I - Survey Questionnaire

The questionnaire below was asked of participants following confirmation of informed consent using an online platform (Qualtrics) compatible with desktop and mobile devices.

Section 1 of 3. Your Occupancy Within Your Residential Property BEFORE the COVID-19 Pandemic reached Canada.
Please answer the following questions about how much time ***you personally*** *spent in your property BEFORE the widespread changes caused by the COVID-19 pandemic in Canada (e.g. for most Canadians this would be the period* ***before March 1, 2020****).*

PLEASE NOTE: It is ok to generalize your responses based on your general experiences and habits in the previous 1-5 years before March 1, 2020.

Click the yellow right-facing arrow below to continue.

NOTE: All questions containing "BEFORE*" mention "*e.g. for most Canadians this would be the period before March 1, 2020."

1. **BEFORE*** the COVID-19 pandemic reached Canada, what was your status in terms of employment or enrollment in education**?
*** We are gathering this information as it is essential to understanding how radon exposure may have changed during the pandemic in reference to Canadians variably in school, work, or alternative scenarios.*
If answer a -> Go to S1Q2 | If answer b to e -> Go to S1Q4
   a. Full-time or part-time employed and/or enrolled in education**
      **These activities may take place at an office, school, or from your property. The next question(s) will ask you to tell us about where these are taking place.
   b. Unemployed and/or not enrolled in education or on temporary leave
   c. Retired
   d. On long-term leave (including parental leaves, medical leaves, disability, etc.)
   e. Prefer not to say

2. Which category best represents the industry / sector** that you were employed in **BEFORE*** the COVID-19 pandemic reached Canada? Go to S1Q3
*** We are gathering this information as it is essential to understanding how radon exposure may have changed during the pandemic in reference to different sectors of the Canadian workforce. Please note, these categories are derived from Statistics Canada's North American Industry Classification System (NAICS) 2017 Version 3.0*

   a. Not applicable; I am a full-time student
   b. Agriculture, forestry, fishing and hunting
   c. Mining, quarrying, and oil and gas extraction
   d. Utilities
   e. Construction

f. Manufacturing
g. Wholesale trade
h. Retail trade
i. Transportation and warehousing
j. Information and cultural industries
k. Finance and insurance
l. Real estate and rental and leasing
m. Professional, scientific, and technical services
n. Management of companies and enterprises
o. Administrative and support, waste management and remediation services
p. Educational services
q. Health care and social assistance
r. Arts, entertainment and recreation
s. Accommodation and food services
t. Other services (except public administration)
u. Public administration

3. In as simple terms possible, please indicate your broad <u>job title</u>** **BEFORE*** the COVID-19 pandemic reached Canada. For example, this might be something like "nurse," "accountant," "salesperson," "scientist," "realtor," "farmer," "social worker," "artist," "entertainer," or "teacher."

** *We are gathering this information as it is essential to understanding how radon exposure may have changed during the pandemic in reference to different specific jobs within the Canadian workforce, independent of what sector you are in.*

Go to S1Q5

____

4. **BEFORE*** the COVID-19 pandemic reached Canada, how many *hours per day* in each season did you typically spend: Go to S2Q1

	**INSIDE** your residence (tested by Evict Radon)	**INSIDE** a different residence (vacation home, boutique hotel, friend's home etc.)	**INSIDE** a non-residential building (an office, store, business, school, large hotel etc., including commuting via underground train)	**OUTDOORS** (including camping and commuting via car, plane, bus, bike, over-land train, or walking)	Total
Winter (Dec, Jan, Feb)	hours per day	hours per day	hours per day	hours per day	hours per day
Spring (Mar, Apr, May)	hours per day	hours per day	hours per day	hours per day	hours per day
Summer (Jun, Jul, Aug)	hours per day	hours per day	hours per day	hours per day	hours per day

Fall (Sep, Oct, Nov)	hours per day	hours per day	hours per day	hours per day	hours per day

5. **BEFORE*** the COVID-19 pandemic reached Canada, on average how many *days per week* did you typically spend regularly attending work or school in each season (whether this was done in your residence or in another building)? Go to S1Q6

a. days per week in "Winter" (Dec, Jan, Feb)
b. days per week in "Spring" (Mar, Apr, May)
c. days per week in "Summer" (Jun, Jul, Aug)
d. days per week in "Fall" (Sep, Oct, Nov)

6. **BEFORE*** the COVID-19 pandemic reached Canada, approximately how many *hours per day* during a work or school day in each season did you typically spend: Go to S1Q7

	**INSIDE** your residence (tested by Evict Radon)	**INSIDE** a different residence (vacation home, boutique hotel, friend's home etc.)	**INSIDE** a non-residential building (an office, store, business, school, large hotel etc., including commuting via underground train)	**OUTDOORS** (including camping and commuting via car, plane, bus, bike, over-land train, or walking)	Total
Winter (Dec, Jan, Feb)	hours per day	hours per day	hours per day	hours per day	hours per day
Spring (Mar, Apr, May)	hours per day	hours per day	hours per day	hours per day	hours per day
Summer (Jun, Jul, Aug)	hours per day	hours per day	hours per day	hours per day	hours per day
Fall (Sep, Oct, Nov)	hours per day	hours per day	hours per day	hours per day	hours per day

7. **BEFORE*** the COVID-19 pandemic reached Canada, approximately how many *hours per day* during a weekend day or holiday in each season did you typically spend: Go to S2Q1

	**INSIDE** your residence (tested by Evict Radon)	**INSIDE** a different residence (vacation home, boutique hotel, friend's home etc.)	**INSIDE** a non-residential building (an office, store, business, school, large hotel etc., including commuting via underground train)	**OUTDOORS** (including camping and commuting via car, plane, bus, bike, over-land train, or walking)	Total
Winter (Dec, Jan, Feb)	hours per day	hours per day	hours per day	hours per day	hours per day

Spring (Mar, Apr, May)	hours per day	hours per day	hours per day	hours per day	hours per day
Summer (Jun, Jul, Aug)	hours per day	hours per day	hours per day	hours per day	hours per day
Fall (Sep, Oct, Nov)	hours per day	hours per day	hours per day	hours per day	hours per day

----

Section 2 of 3. Your Occupancy Within Your Residential Property During the FIRST TO THIRD WAVES* of the COVID-19 Pandemic in Canada.

*Please answer the following questions about how much time **you personally** spent in your property during the FIRST TO THIRD WAVES of the widespread changes caused by the COVID-19 pandemic in Canada (i.e. during the period between **March 1, 2020 and May 31, 2021**).*

PLEASE NOTE: It is ok to generalize your responses based on your experience across the entire period.

Click the yellow right-facing arrow below to continue.

NOTE: All questions containing "During the FIRST TO THIRD WAVES*" mention "*i.e. during the period between March 1, 2020 and May 31, 2021."

1. During the **FIRST TO THIRD WAVES*** of the COVID-19 pandemic in Canada, has your status** in terms of employment and/or enrollment in education changed in any way? If answer a or c -> Go to S2Q2 | If answer b AND S1Q1 a -> Go to S2Q3 | If answer b AND S1Q1 b to e -> Go to S2Q5

**Please note: Your status refers to the amount of time you spent working or learning at any location (even if this was your property).

  a. Yes
  b. No
  c. Prefer not to say

2. During the **FIRST TO THIRD WAVES*** of the COVID-19 pandemic in Canada, what was your status in terms of employment or enrollment in education**?

*** We are gathering this information as it is essential to understanding how radon exposure may have changed during the pandemic in reference to Canadians variably in school, work, or alternative scenarios.*

If answer a -> Go to S2Q3 | If answer b to e -> Go to S2Q5

  a. Full-time or part-time employed and/or enrolled in education**

     **These activities may take place at an office, school, or from your property. The next question(s) will ask you to tell us about where these are taking place.

  b. Unemployed and/or not enrolled in education or on temporary leave
  c. Retired

d. On long-term leave (including parental leaves, medical leaves, disability, etc.)
e. Prefer not to say

3. Which category best represents the industry / sector** that you were employed in during the **FIRST TO THIRD WAVES*** of the COVID-19 pandemic in Canada? Go to S2Q4

*** We are gathering this information as it is essential to understanding how radon exposure may have changed during the pandemic in reference to different sectors of the Canadian workforce.* Please note, these categories are derived from Statistics Canada's North American Industry Classification System (NAICS) 2017 Version 3.0

a. Not applicable; I am a full-time student
b. Same as **BEFORE** the COVID-19 pandemic reached Canada
c. Agriculture, forestry, fishing and hunting
d. Mining, quarrying, and oil and gas extraction
e. Utilities
f. Construction
g. Manufacturing
h. Wholesale trade
i. Retail trade
j. Transportation and warehousing
k. Information and cultural industries
l. Finance and insurance
m. Real estate and rental and leasing
n. Professional, scientific, and technical services
o. Management of companies and enterprises
p. Administrative and support, waste management and remediation services
q. Educational services
r. Health care and social assistance
s. Arts, entertainment and recreation
t. Accommodation and food services
u. Other services (except public administration)
v. Public administration

4. In as simple terms possible, please indicate your broad job title* during the **FIRST TO THIRD WAVES*** of the COVID-19 pandemic in Canada. For example, this might be something like "nurse," "accountant," "salesperson," "scientist," "realtor," "farmer," "social worker," "artist," "entertainer," or "teacher."

** We are gathering this information as it is essential to understanding how radon exposure may have changed during the pandemic in reference to different specific jobs within the Canadian workforce, independent of what sector you are in.*

Go to S2Q6

____

5. During the **FIRST TO THIRD WAVES*** of the COVID-19 pandemic in Canada, how many *hours per day* in each season did you typically spend: Go to S3Q1

	**INSIDE** your residence	**INSIDE** a different residence	**INSIDE** a non-residential building (an office, store, business,	**OUTDOORS** (including camping and	Total

	(tested by Evict Radon)	(vacation home, boutique hotel, friend's home etc.)	school, large hotel etc., including commuting via underground train)	commuting via car, plane, bus, bike, over-land train, or walking)	
"Spring #1" Mar, Apr, May of 2020	hours per day	hours per day	hours per day	hours per day	hours per day
"Summer" Jun, Jul, Aug of 2020	hours per day	hours per day	hours per day	hours per day	hours per day
"Fall" Sep, Oct, Nov of 2020	hours per day	hours per day	hours per day	hours per day	hours per day
"Winter" Dec 2020, Jan 2021, Feb 2021	hours per day	hours per day	hours per day	hours per day	hours per day
"Spring #2" Mar, Apr, May of 2021	hours per day	hours per day	hours per day	hours per day	hours per day

6. During the **FIRST TO THIRD WAVES*** of the COVID-19 pandemic in Canada, on average how many *days per week* did you typically spend regularly attending work or school in each season (whether this was done in your residence or in another building)? Go to S2Q7

a. days per week in "Spring #1" (Mar, Apr, May of 2020)
b. days per week in "Summer" (Jun, Jul, Aug of 2020)
c. days per week in "Fall" (Sept, Oct, Nov of 2020)
d. days per week in "Winter" (Dec 2020, Jan 2021, Feb 2021)
e. days per week in "Spring #2" (Mar, Apr, May of 2021)

7. During the **FIRST TO THIRD WAVES*** of the COVID-19 pandemic in Canada, approximately how many *hours per day* during a work or school day in each season did you typically spend: Go to S2Q8

	**INSIDE** your residence (tested by Evict Radon)	**INSIDE** a different residence (vacation home, boutique hotel, friend's home etc.)	**INSIDE** a non-residential building (an office, store, business, school, large hotel etc., including commuting via underground train)	**OUTDOORS** (including camping and commuting via car, plane, bus, bike, over-land train, or walking)	Total

"Spring #1" Mar, Apr, May of 2020	hours per day	hours per day	hours per day	hours per day	hours per day
"Summer" Jun, Jul, Aug of 2020	hours per day	hours per day	hours per day	hours per day	hours per day
"Fall" Sep, Oct, Nov of 2020	hours per day	hours per day	hours per day	hours per day	hours per day
"Winter" Dec 2020, Jan 2021, Feb 2021	hours per day	hours per day	hours per day	hours per day	hours per day
"Spring #2" Mar, Apr, May of 2021	hours per day	hours per day	hours per day	hours per day	hours per day

8. During the **FIRST TO THIRD WAVES*** of the COVID-19 pandemic in Canada, approximately how many *hours per day* during a weekend day or holiday in each season did you typically spend: Go to S3Q1

	**INSIDE** your residence (tested by Evict Radon)	**INSIDE** a different residence (vacation home, boutique hotel, friend's home etc.)	**INSIDE** a non-residential building (an office, store, business, school, large hotel etc., including commuting via underground train)	**OUTDOORS** (including camping and commuting via car, plane, bus, bike, over-land train, or walking)	Total
"Spring #1" Mar, Apr, May of 2020	hours per day	hours per day	hours per day	hours per day	hours per day
"Summer" Jun, Jul, Aug of 2020	hours per day	hours per day	hours per day	hours per day	hours per day
"Fall" Sep, Oct, Nov of 2020	hours per day	hours per day	hours per day	hours per day	hours per day
"Winter" Dec 2020, Jan 2021, Feb 2021	hours per day	hours per day	hours per day	hours per day	hours per day

"Spring #2" Mar, Apr, May of 2021	hours per day	hours per day	hours per day	hours per day	hours per day

----

Section 3 of 3. Demographic Information. *Please answer the following demographic questions, which will help us understand how the COVID-19 pandemic has altered cancer risk in Canada, and for whom.*

We hope that you are able to share some of this vital information with our cancer prevention team, knowing that we will only ever use this for research into improving radon protection from a public health (non-commercial) perspective. We will also never share your information with any third party, and it will only ever be used as an aggregate dataset – meaning you can never ever be personally identified.

Click the yellow right-facing arrow below to begin this section.

1. What is your age* (as of this year)? Go to S3Q2

* *We are gathering this information as we have recently discovered that age a relevant contributor to risk for radon exposure, and also how people are impacted by the COVID-19 pandemic. If you are not comfortable giving your exact age, please provide a number rounded to the nearest 5-year period of your life (e.g. if you are 51, you could put "50". If you are 24, you could put "25" and so forth).*

Number (between 18-125 only)

2. What sex* were you assigned at birth? Go to S3Q3

* *We are gathering this information as biological sex is a relevant contributor to how radon impacts the human body, as well as risk for radon-induced lung cancer. The next question will ask you to indicate your gender identity.*

a. Male
b. Female

3. What is your current gender identity*? Please select all that apply. Go to S3Q4

* *We are gathering this information as gender is a relevant social determinant for risk of exposure to radon, as well as how people have been impacted during the COVID-19 pandemic.*

a. Male
b. Female
c. Transgender
d. Genderqueer/gender non-conforming
e. Nonbinary
f. Prefer to self-describe: _______
g. Prefer not to say

4. Which broad category would you say best identifies the general regional context* of your current residence? **We are gathering this information as we need to evaluate whether pandemic-related changes in radon exposure may differ depending on how "rural or urban" your household identifies, potentially making a case for added support for those disproportionately effected in a negative manner.* Go to S3Q5

a. An urban, metropolitan centre (my residence is located in an area of higher household density, towards the centre of a city, large town, etc.)

b. A suburban metropolitan area (my residence is located in an area of medium household density, surrounding an urban metropolitan centre)
c. A rural area (my residence is located in an area of lower household density, such as a smaller towns, villages, or hamlets that are not classified as suburbs of any major urban centre)
d. A First Nations / Indigenous community (of any description)
e. A rural commune (of any description)
f. An isolated residence (my primary residence is a single, stand alone property with no nearby neighbours – this may, for example, include some farm homes)
g. Unsure or prefer not to say

5. Approximately how long have you lived in Canada*?

* *We are gathering this information as the relative amount of time a person has lived in a given country is a relevant social determinant for health system access, risk awareness, as well occupancy trends within the residential environment. Knowing this will help us determine if pandemic-related changes to radon exposure may differ across different groups, potentially making a case for support for those disproportionately effected in a negative manner.* Go to S3Q6

a. I was born in Canada
b. I immigrated/moved to Canada within the last 5 years
c. I immigrated/moved to Canada between 6 and 10 years ago
d. I immigrated/moved to Canada more than 10 years ago
a. Unsure or prefer not to say

6. What was your highest level of formal education* as of this date? Go to S3Q7

* *We are gathering this information as the highest level of formal education a person has reached is a relevant determinant for Socioeconomic status, which impacts health system access, risk awareness, as well occupancy trends within the residential environment. Knowing this will help us determine if pandemic-related changes to radon exposure may differ across different groups, potentially making a case for added support for those disproportionately effected in a negative manner.*

a. No formal education
b. Some primary or secondary school
c. High school diploma
d. Some university/college/technical school
e. Technical certification, completed
f. College/university degree, completed
g. University graduate degree, completed
h. Unsure or prefer not to say

7. What was your approximate, average <u>HOUSEHOLD INCOME</u>* over the past year**?

* *Household income is defined as the combined gross income of all adult members of the same primary residence. Individuals do not have to be related in any way to be considered members of the same household, although, if you have roommates or tenants who you do not know very well / are 'not part of your life', it is ok not to consider them in your response*

** *We are gathering this information as household income is a relevant determinant for socioeconomic status, which impacts health system access, risk awareness, as well occupancy trends within the residential environment. Knowing this will help us determine if pandemic-related changes to radon exposure may differ across different groups, potentially making a case for added support for those disproportionately effected in a negative manner.* Go to S3Q8

a. No income or loss
b. Less than $10,000

c. $10,001 to $20,000
d. $20,001 to $30,000
e. $30,001 to $40,000
f. $40,001 to $50,000
g. $50,001 to $60,000
h. $60,001 to $80,000
i. $80,001 to $100,000
j. $100,001 to $200,000
k. More than $200,000
l. Unsure or prefer not to say

8. As of this date, and including yourself in the count, how many adults (18 or more years old) live regularly in your residence? Go to S3Q9

adults total

9. As of this date, how many minors (0 – 17 years old) live regularly in your residence? Go to END

minors total

We thank-you for your time spent taking this survey. Your response has been recorded.

SECTION II – Examples of Microinfluencer Posts

The screenshots below are a representative sample of Instagram social media platform posts from microinfluencers that were contracted to deliver radon awareness information in our case study. Subscriber identities are obscured by black boxes to preserve privacy.

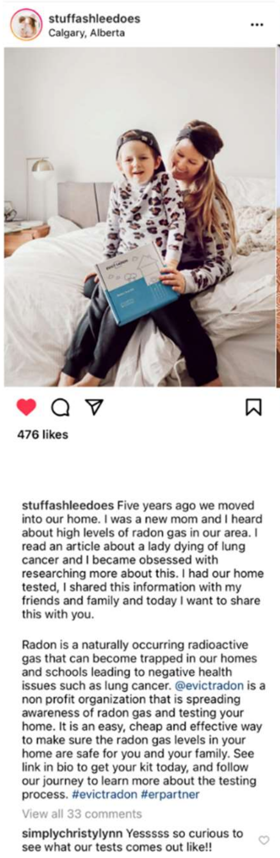

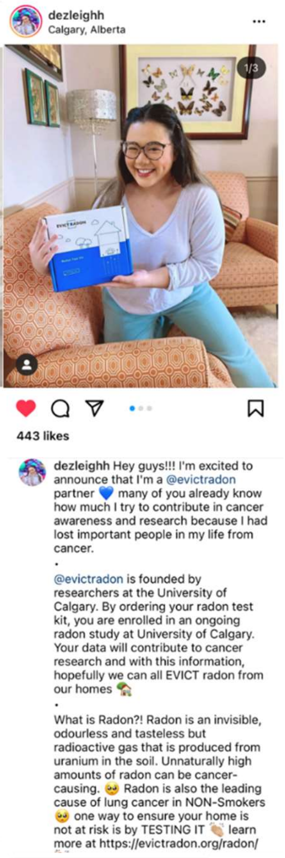

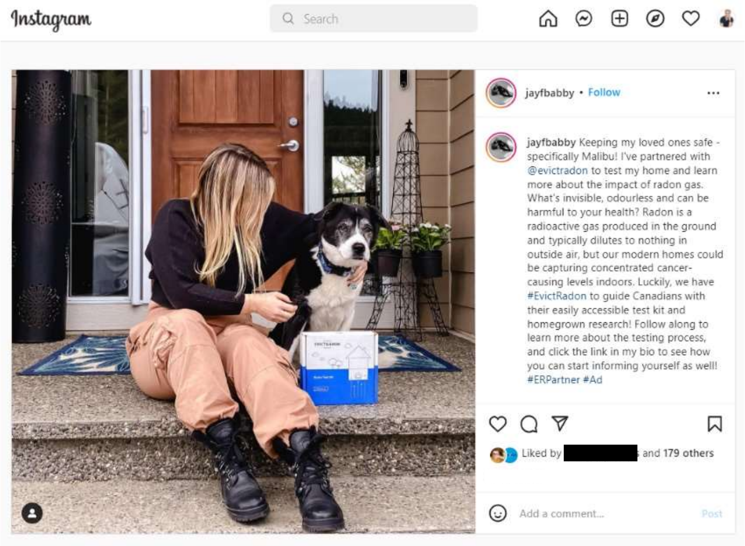

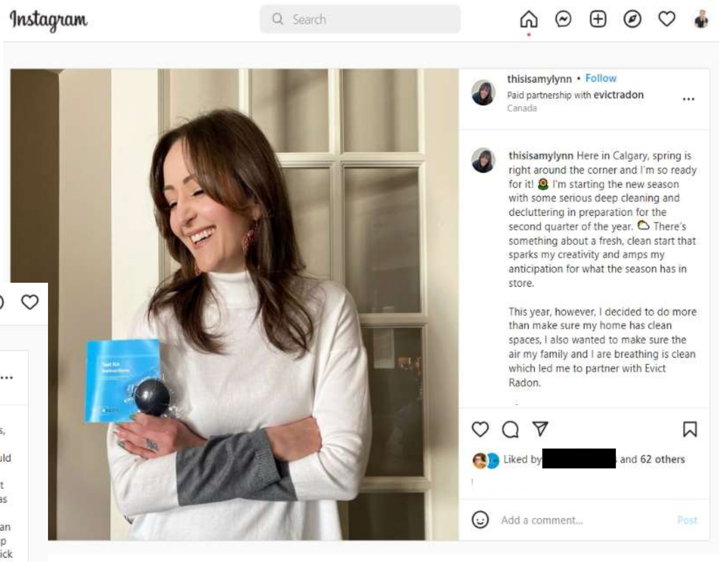

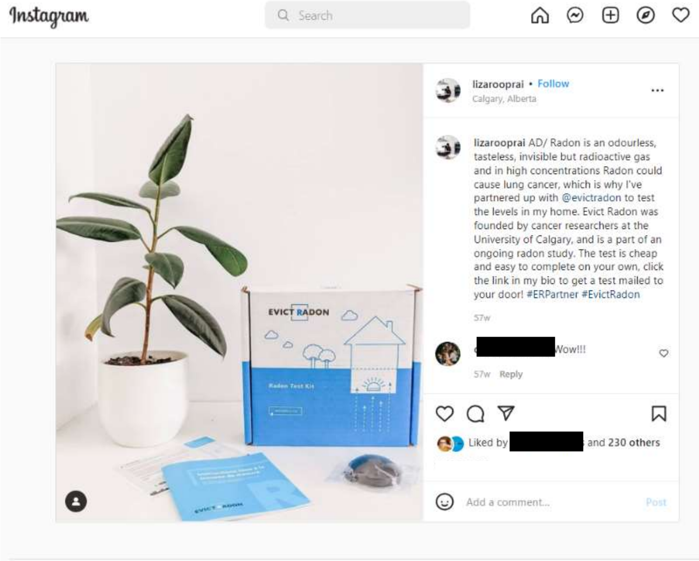

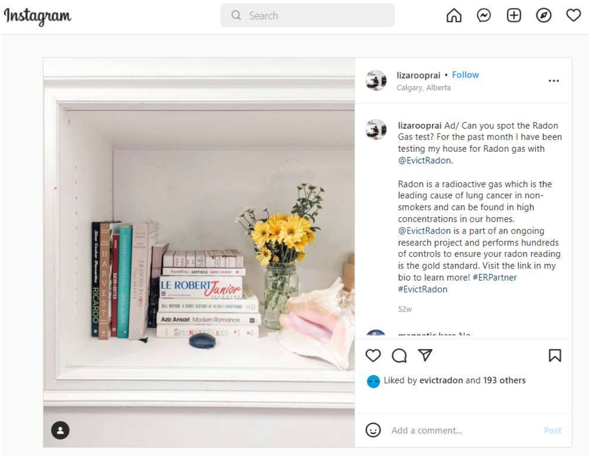
Instagram
Search
lizarooprai • Follow
Calgary, Alberta
lizarooprai Ad/ Can you spot the Radon Gas test? For the past month I have been testing my house for Radon gas with @EvictRadon.
Radon is a radioactive gas which is the leading cause of lung cancer in non-smokers and can be found in high concentrations in our homes. @EvictRadon is a part of an ongoing research project and performs hundreds of controls to ensure your radon reading is the gold standard. Visit the link in my bio to learn more! #ERPartner #EvictRadon
52w
Liked by evictradon and 193 others
Add a comment...
Post

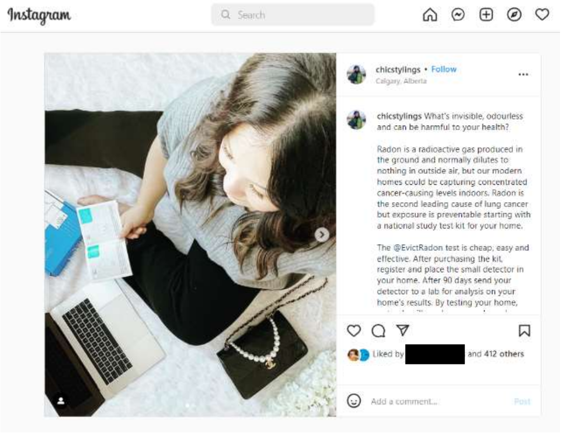
Instagram
Search
chicstylings • Follow
Calgary, Alberta
chicstylings What's invisible, odourless and can be harmful to your health?
Radon is a radioactive gas produced in the ground and normally dilutes to nothing in outside air, but our modern homes could be capturing concentrated cancer-causing levels indoors. Radon is the second leading cause of lung cancer but exposure is preventable starting with a national study test kit for your home.
The @EvictRadon test is cheap, easy and effective. After purchasing the kit, register and place the small detector in your home. After 90 days send your detector to a lab for analysis on your home's results. By testing your home,
Liked by [redacted] and 412 others
Add a comment...
Post

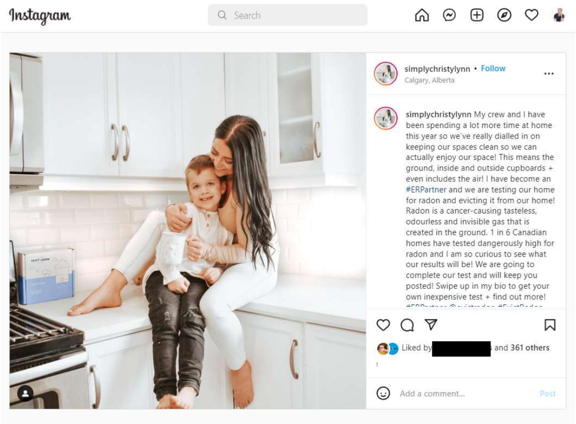
Instagram
Search
simplychristylynn • Follow
Calgary, Alberta
simplychristylynn My crew and I have been spending a lot more time at home this year so we've really dialled in on keeping our spaces clean so we can actually enjoy our space! This means the ground, inside and outside cupboards + even includes the air! I have become an #ERPartner and we are testing our home for radon and evicting it from our home! Radon is a cancer-causing tasteless, odourless and invisible gas that is created in the ground. 1 in 6 Canadian homes have tested dangerously high for radon and I am so curious to see what our results will be! We are going to complete our test and will keep you posted! Swipe up in my bio to get your own inexpensive test + find out more!
Liked by [redacted] and 361 others
Add a comment...
Post

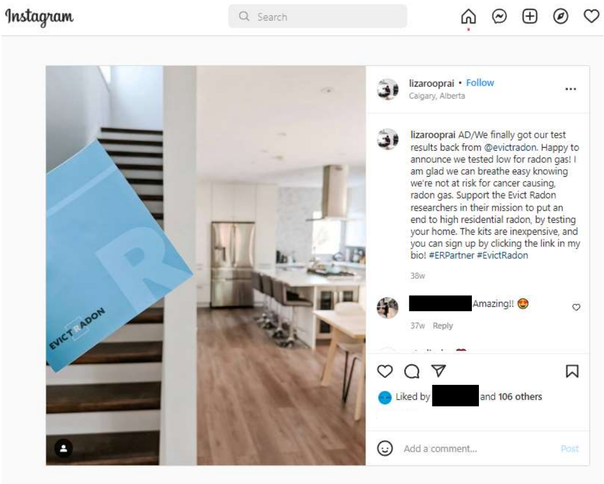
Instagram
Search
lizarooprai • Follow
Calgary, Alberta
lizarooprai AD/We finally got our test results back from @evictradon. Happy to announce we tested low for radon gas! I am glad we can breathe easy knowing we're not at risk for cancer causing, radon gas. Support the Evict Radon researchers in their mission to put an end to high residential radon, by testing your home. The kits are inexpensive, and you can sign up by clicking the link in my bio! #ERPartner #EvictRadon
38w
[redacted] Amazing!! 😍
37w Reply
Liked by [redacted] and 106 others
Add a comment...
Post

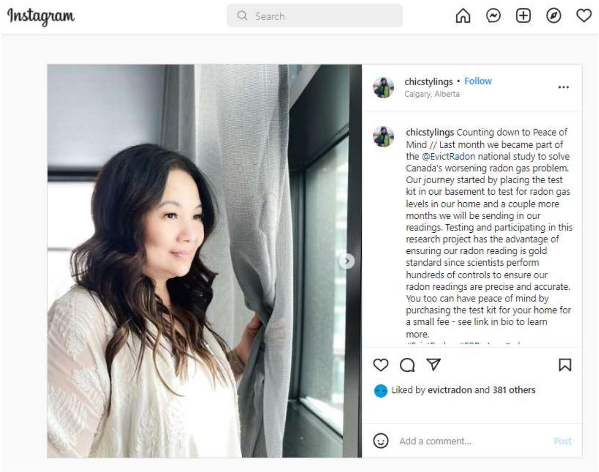
Instagram
Search
chicstylings • Follow
Calgary, Alberta
chicstylings Counting down to Peace of Mind // Last month we became part of the @EvictRadon national study to solve Canada's worsening radon gas problem. Our journey started by placing the test kit in our basement to test for radon gas levels in our home and a couple more months we will be sending in our readings. Testing and participating in this research project has the advantage of ensuring our radon reading is gold standard since scientists perform hundreds of controls to ensure our radon readings are precise and accurate. You too can have peace of mind by purchasing the test kit for your home for a small fee - see link in bio to learn more.
Liked by evictradon and 381 others
Add a comment...
Post

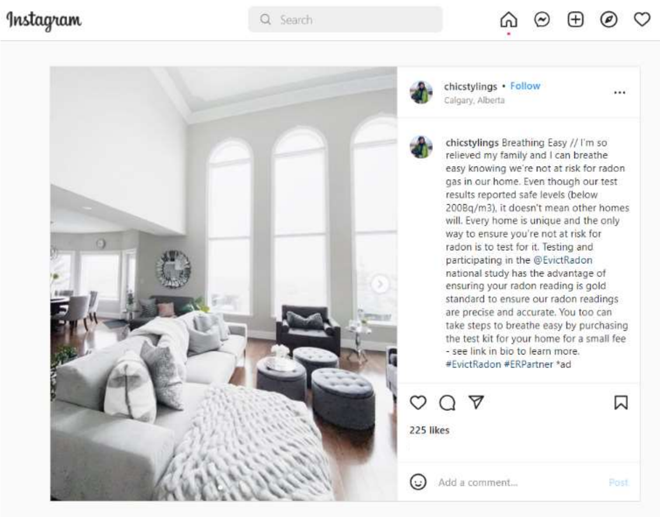
Instagram
Search
chicstylings • Follow
Calgary, Alberta
chicstylings Breathing Easy // I'm so relieved my family and I can breathe easy knowing we're not at risk for radon gas in our home. Even though our test results reported safe levels (below 200Bq/m3), it doesn't mean other homes will. Every home is unique and the only way to ensure you're not at risk for radon is to test for it. Testing and participating in the @EvictRadon national study has the advantage of ensuring your radon reading is gold standard to ensure our radon readings are precise and accurate. You too can take steps to breathe easy by purchasing the test kit for your home for a small fee - see link in bio to learn more. #EvictRadon #ERPartner *ad
225 likes
Add a comment...
Post

SECTION III – Further description of Interaction Indices

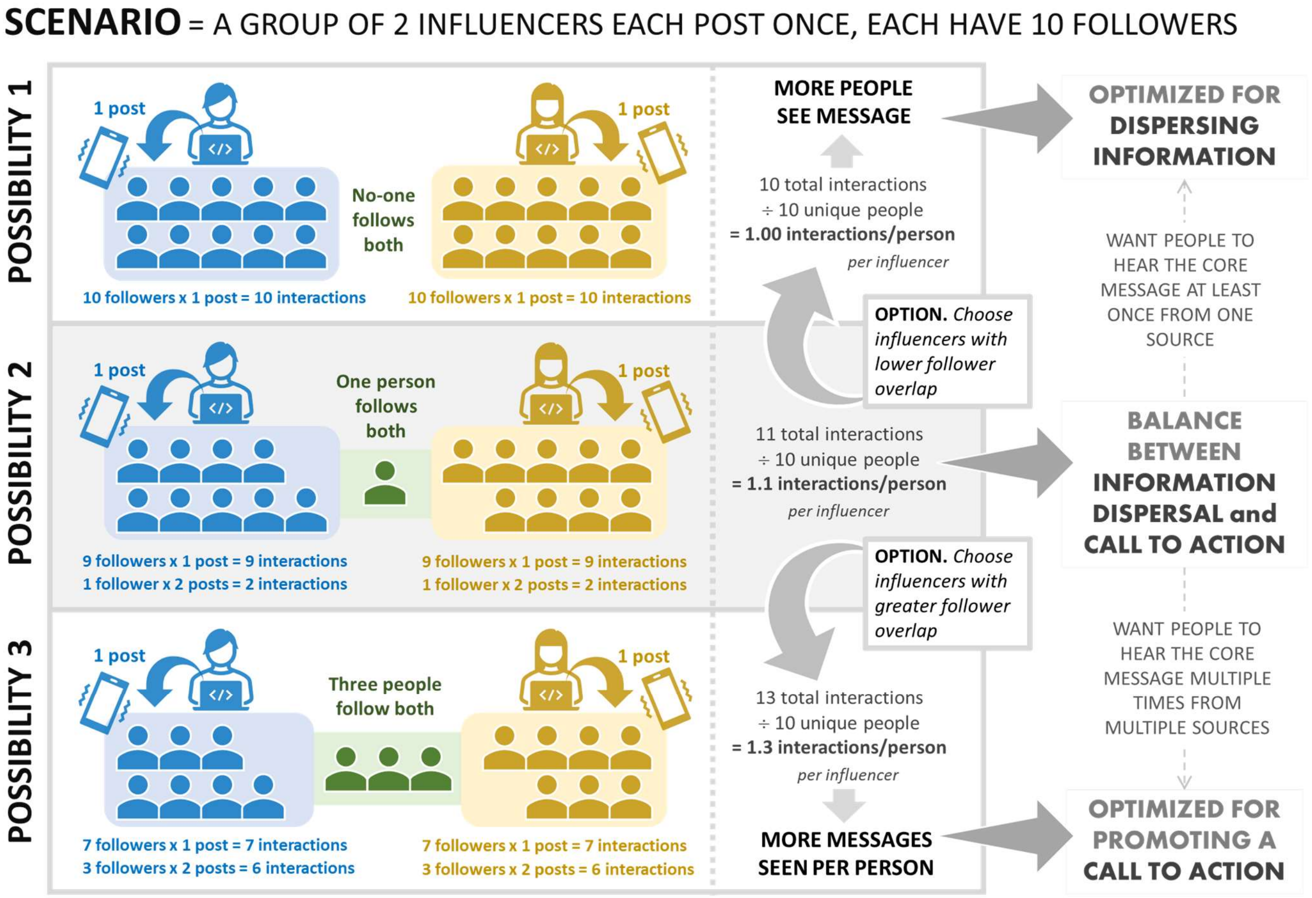

CALCULATING an influencer group's INTERACTION INDEX

The number of times a person in the target audience is likely to receive a unique message, per occasion that the influencers within the chosen group each release a post
